# Healthcare utilisation among low-income individuals affected by violence in Rio de Janeiro, Brazil: a retrospective cohort study

**DOI:** 10.1186/s12889-025-24567-2

**Published:** 2025-10-16

**Authors:** Sophia Medeiros, Julia Guerra, Vinicius Peçanha, Joana Monteiro, Rudi Rocha, Christopher Millett, Thomas Hone

**Affiliations:** 1 Public Health Policy Evaluation Unit, Department of Primary Care and Public Health, School of Public Health, Imperial College London, London, UK; 2 Instituto de Estudos para Políticas de Saúde (IEPS), São Paulo, Brazil; 3 Escola de Administração de Empresas de São Paulo da Fundação Getulio Vargas (FGV EAESP), São Paulo, Brazil; 4 Escola Brasileira de Administração Pública e de Empresas da Fundação Getulio Vargas (FGV EBAPE), Rio de Janeiro, Brazil; 5 Public Health Research Center, Comprehensive Health Research Center, NOVA National School of Public Health, NOVA University Lisbon, Lisbon, Portugal

**Keywords:** Violence, Health outcomes, Primary healthcare, Healthcare utilisation, Hospitalisation, Rio de janeiro, Brazil, Urban, LMIC, Coarsened exact matching

## Abstract

**Background:**

Violence is associated with adverse health outcomes and affects healthcare utilisation, but there is limited robust evidence on this relationship in the context of violence in urban environments. This study examines subsequent patterns of healthcare utilisation by individuals who accessed healthcare as a result of experiencing violence in Rio de Janeiro, Brazil.

**Methods:**

A retrospective cohort analysis was conducted on 529,219 low-income individuals from January 2010 to December 2016. Electronic medical records were screened for ICD-10 and primary care codes, and clinical free text identifying individuals who used healthcare due to violence. Control individuals (who did not use healthcare due to violence) were identified via coarsened exact matching. Logistic panel regression assessed the association between healthcare use due to violence and subsequent healthcare utilisation.

**Results:**

2,038 individuals used healthcare due to violence. This group were 11% more likely (OR:1.11;95%CI:1.00–1.24;*p* < 0.05) to use PHC in the subsequent 1–3 months after initial healthcare use compared to those not using healthcare due to violence. They were 79% less likely (OR:0.21;95%CI:0.05–0.93;*p* < 0.05) to be Hospitalised at 7–12 months. Female users were 4.25 times (OR:4.25;95%CI:2.14–8.44;*p* < 0.001) and 2.57 times (OR:2.57;95%CI:1.20–5.53;*p* < 0.05) more likely to use PHC for poor pregnancy outcomes in the first 1–3 months and 7–12 months, respectively. PHC use for mental health increased by 50% (OR:1.50;95%CI:1.27–1.77;*p* < 0.005) in the subsequent 1–3 months after initial healthcare use.

**Conclusions:**

Violence was associated with short-term increases in healthcare utilisation among low-income individuals in Rio de Janeiro, Brazil.

**Supplementary Information:**

The online version contains supplementary material available at 10.1186/s12889-025-24567-2.

## Background 

Violence is a major threat to global and human development. Violence is responsible for over 500,000 deaths each year, which is estimated to increase to 610,000 by 2030 [1, 2]. Violence is a persistent public health problem in Brazil [3]. Among Latin American countries, a region with one of the highest Homicide rates globally, Brazil ranks among the highest with a Homicide rate of 30.76 per 100,000 population in 2020 – three times the global average [3, 4]whilst violence is the sixth leading cause of hospital admissions [5]. 

Experiencing violence, including being directly harmed, affected by, or witnessing violence, has been associated with poorer physical and psychological health in numerous studies [6–14]. The most visible, immediate health impacts of violence are injuries and homicides. However, there are long-term effects on both physical and mental health. Existing evidence from the United States and conflict-affected regions including Palestine, Libya, and Afghanistan, suggests that violence increases the risk of chronic illnesses like asthma, cardiovascular disease, cancer, and stroke as well as poor psychological health and birth outcomes [6–9]. Individuals fear accessing healthcare services in violence-afflicted settings which discourages health seeking behaviours and service utilisation [15–18]. On the other hand, some evidence suggests that worsening health status and stress in violent settings may increase health service utilisation. For example, injuries may lead to an increase in emergency room visits and violence-related stress may result in long-term mental health needs, increasing short- and long-term utilisation [8, 19, 20]. 

**Fig. 1:**
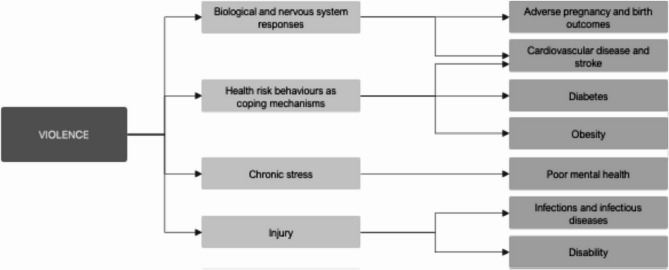
Conceptual framework of the pathways through which violence increases health risk factors. Sources: Scott-Storey [21], Guha-Sapir & van Panhuis [22], and author’s own work. See Appendix 1 for more detail

Violence affects health through multiple pathways that increases risk factors leading to adverse health outcomes. Considering Scott-Storey’s [21] and Guha-Sapir & van Panhuis’ [22] frameworks on violence and health, four key pathways through which violence increases health risks can be identified: biological and nervous system responses, health risk behaviours as coping mechanisms, chronic stress, and injury. These pathways contribute to various health conditions, including adverse pregnancy and birth outcomes, cardiovascular disease and stroke, diabetes, obesity, poor mental health, infections and infectious diseases, and disability (Fig. 1; Appendix 1).

Despite the understanding that violence affects both short- and long-term health, there is little empirical evidence on patterns of healthcare utilisation in those exposed to violence [23–25]. Studies in low- and middle-income countries (LMICs) tend to focus on intimate partner violence and utilisation of maternal care services [26–30]. Most evidence originates from high income countries [31, 32], focusses on violence exposure in childhood and adolescence [11, 12, 15, 17], or is based on self-reported violence and health status [10, 12, 15], with limited investigation on patterns of healthcare utilisation.

The city of Rio de Janeiro is an important setting for this study. An estimated one fifth of Rio de Janeiro’s population live in slums (favelas) where violence can be endemic [33–35]. Individuals living in slums across Latin America frequently experience high levels of violence as a result of drug trafficking, organised crime, gang activity, and police operations. While existing evidence demonstrates associations between violence and unsafe communities, reduced healthcare access, and reduced investment into public services [36, 37], no study has comprehensively explored the impact of violence on healthcare utilisation.

This study quantifies patterns of healthcare utilisation following an intitial healthcare consultation due to violence among low-income individuals in Rio de Janeiro. We combine individual electronic health records and hospitalisation records with socioeconomic and demographic data. This study employs a novel approach by screening clinical free text records—an approach that has been used in high-income countries and shown to offer advantages over structured data alone. It enables the identification of individuals who report or discuss violence, even when it is not recorded as a diagnosis or procedure code, thereby capturing more detailed information and improving research quality and accuracy [38]. In this study we focus on violence that would expectedly occur outside the home and violence which relates to the city and urban environment (i.e. in the community/neighbourhood rather than the domestic environment). Rape and sexual abuse/assault in Brazil is largely interpretable as domestic violence and warrants separate investigation given the distinctly different policy responses, interventions, and advocacy efforts [39, 40]. Therefore, we exclude violence related to rape and sexual abuse/assault from our analyses. We aim to understand the effect of violence, such as physical assault, crime, and gang activity, on the risk of further healthcare use over time by quantifying primary healthcare utilisation and hospitalisation for violence-related health conditions previously shown to be adversely impacted by violence exposure.

## Methods

### Study design

This is a retrospective cohort analysis of primary healthcare-registered users exposed to violence and their matched controls with linked welfare, primary healthcare (PHC), and hospitalisation records.

### Study population

Our study population is a derived from a larger cohort of 3,148,383 individuals registered with PHC clinics in Rio de Janeiro. We obtained information on individuals from the *Ficha de Cadastro Domiciliar e Territorial* (Household and Territorial Registration Form; Ficha A) from the Brazilian Ministry of Health [41]. These PHC registration records were linked to PHC electronic medical records from the Primary Care Assessment Tool (PCAT) dataset to identify PHC users and public hospitalisation records (*Sistema de Informações Hospitalares*; SIH), all covering the period from 1 January 2010 to 31 December 2016. The datasets were linked via a combination of deterministic and probabilistic approaches, with details published elsewhere [42]. 

We identified PHC users exposed to violence, and using coarsened exact matching identified control individuals not exposed to violence, resulting in a final study population of 529,219 individuals. PHC services in Rio de Janeiro are concentrated in low-income areas, meaning the study population largely consisted of lower-income individuals living in poorer neighbourhoods at greater risk of violence.

### Matching

We exploited one-to-many coarsened exact matching (CEM) with no repeats to reduce potential unobserved confounding and ensure covariate balance. Covariate balance was assessed using the linear 1 (L1) statistic [43]. Exposed individuals who did not have matches were dropped from the dataset (*n* = 11). Exposure status was determined prior to conducting CEM (on the original 3.1 million sample) to prevent bias in group classification and ensure that the matching process was based solely on confounders. Nine covariates were used for matching: micro area code (a geographical administrative unit responsible for local health services to account for potential differences in healthcare access), sex, race/skin colour, education, age group, household income, private healthcare insurance, whether an individual’s family claimed welfare, and whether an individual used healthcare (PHC or hospitalisation) in the study period. Information on the sample size and covariate balancing pre- and post-matching can be found in Appendix 4.

### Exposure

Individuals were considered exposed to violence if they used PHC or were hospitalised due to violence. This was classified according to International Classification of Disease (ICD-10) codes and the International Classification of Primary Care (ICPC) code Z25 (assault/harmful event problem) (Table 1) – for either primary or secondary causes. We also searched clinical free text in PHC consultation notes, detailed in Appendix 2. For each individual, we obtained the month-year of their first healthcare use for violence and used this to construct our exposure variable.

**Table 1:** International classification of disease (ICD-10) and international classification of primary care (ICPC) codes to identify cases of violence in electronic medical records

VIOLENCE SUB-TYPES	CODES
Perpetrated with object	X99, Y00
Firearms	X93-X95, W32-W34, Y22-Y24
Drugs, chemicals, explosives	X85-X90, X96-X98
Suffocation	X91-X92
Intentional action (pushing, crashing)	Y01-Y03
Bodily force and physical violence	Y04, R45.6
Other maltreatment by unknown person	Y07.2, Y07.3
Assault by other means	Y08-Y09
Other intentional injuries	Y35
Sequelae of assault and other intentional injuries	Y87.1, Y89
Assault/harmful event problem	Z25*
Examination and observation following other inflicted injury	Z04.5
Victim of crime and terrorism – victim of torture	Z65.4
Exposure to disaster, war and other hostilities	Z65.5

Sources: World Health Organization [44], Romeo et al., [45] Clery et al., [46] Reynolds [47], and author’s own work

*ICPC code. All other codes refer to ICD-10

### Outcomes

Two outcomes were analysed: PHC consultations and hospital admissions. These outcomes were considered for conditions previously shown to be adversely impacted by violence exposure (see conceptual framework; Fig. 1). Health conditions were grouped into five categories according to ICD-10 and ICPC codes: adverse pregnancy and birth outcomes; cardiovascular disease and stroke, diabetes, and obesity; poor mental health; infections and infectious diseases; and disability (Appendix 3) based on clinical presentation. Both primary and secondary causes of PHC utilisation and hospitalisations were used to identify outcomes.

### Covariates

Individual-level covariates, obtained from PHC registration and utilisation records, included race (based on self-reported skin colour in Brazil – Black, White, Pardo [mixed race], Amarelo [Asian], Indigenous, other or unknown [missing]); sex (male, female); highest educational attainment (none/pre-school/literacy class, elementary school, high school or higher education, none reported [missing]); and age group in years (0–18, 19–34, 35–54, 55–64, 65+). Household-level covariates, obtained from Ficha A, included household income (some salary reported to less than full minimum salary, more than one minimum salary but less than two minimum salaries, two minimum salaries or more, no salary reported [unknown or missing]); private healthcare insurance (yes, no); and whether the family receives Bolsa Família—a conditional cash transfer programme for families earning below US$70 per day (yes, no).

Covariates were selected to control for any potential confounders between healthcare use due to violence (exposure) and future healthcare use (outcomes) in sensitivity analyses. Individuals of lower-income, lower educational attainment, and Black/Pardo Brazilians often have limited access to healthcare, influencing health outcomes [48–50]. Additionally, these individuals are more likely to reside in the neighbourhoods with high levels of poverty and crime, increasing the risk of experiencing or being affected by violence [51–54]. 

### Dataset

An individual-level panel was constructed at the month-year level. Outcomes (PHC consultations or hospitalisations for selected health conditions) were observed for individuals from 1 January 2010 to 31 December 2016. Individuals’ start time was defined as either (i) the start of the observation period (1 January 2010) if they were born before 1 January 2010 and PHC registered before 1 January 2010 or (ii) their registration date if they were born after 1 January 2010 and PHC registered before 31 December 2016. Individuals’ end date was defined as either (i) their date of death or (ii) the end of the observation period (31 December 2016).

### Statistical analyses

The total number of PHC consultations and hospitalisations for conditions previously shown to be adversely impacted by violence exposure were reported, overall and by health condition for individuals who used healthcare due to violence and their matched controls. Cohort characteristics, person-years and crude rates of PHC utilisation and hospitalisation of the study population were reported.

Firstly, separate logistic panel regressions were conducted for PHC utilisation and hospitalisation outcomes with a categorical exposure variable representing time since using healthcare due to violence (0 months or did not use healthcare due to violence, 1–3 months, 4–6 months, 7–12 months, over twelve months). For models on hospitalisations, we additionally adjusted for PHC utilisation. Secondly, separate panel logistic regression models were conducted stratified by specific health conditions to understand which health conditions may be driving utilisation. Only PHC outcome regression results were analysed by condition due to low numbers in hospital admissions. Additional two-way interactions between the categorical violence exposure variable and sex, race/skin colour, education level, and household income, were tested to explore socioeconomic inequalities in the associations between violence and PHC utilisation. This analysis aims to identify whether certain groups are disproportionately affected by violence in terms of their healthcare utilisation [48–54]. 

To address the potential for unobserved heterogeneity in individual characteristics (e.g., baseline health status or socioeconomic factors) to bias our estimates, we conducted a fixed-effects analysis. A dummy variable was included for each individual, effectively controlling for all time-invariant characteristics and accounting for unobservable factors that do not change over time. All regression models were clustered at the individual level, and incorporated individual, month, and year fixed effects. Regression models assessing healthcare use for adverse pregnancy and birth outcomes were restricted to females. As all variables were expressed categorically, effect estimates were interpreted as the ratio of the category of interest and the baseline category. Adjusted odds ratios (aORs) or odds ratios (ORs) with 95% confidence intervals (CIs) and p-values were reported. All analyses were performed in Stata V.18.1.

### Sensitivity analyses

To evaluate the robustness of our findings, we conducted sensitivity analyses using alternative model specifications. First, to test the assumption that there may be unobserved heterogeneity across individuals, we used a random-effects logistic regression model which adjusted for differences between individuals and changes within individuals over time. It also allowed us to include individuals who did not have any PHC use or hospitalisation. Second, to test the assumption that fixed-effects logistic regression might not fully address the potential for robust standard errors, we employed a fixed-effects Poisson regression model. This model controlled for all time-invariant individual characteristics while enabling the calculation of robust standard errors. Finally, to test the assumption that correlations within repeated measures of individuals over time could affect the results, we used generalized estimating equations (GEE) with a binomial family and logit link to estimate population-averaged associations. GEE accounted for these correlations, providing a broader perspective on the observed relationships.

## Results

We identified 2,038 individuals who used healthcare due to violence during the study period and selected 527,181 matched controls, representing 1,468,225 person-years of observation (Table 2). The mean number of person-years of observation was 2.77. Over 70% of the study population were female (377,087; 71.3%). By race/skin colour, most of the sample were Pardo (mixed race) (324,583; 61.3%) or White (157,222; 29.7%). Most individuals had either elementary school (216,733; 41.0%) or high school or higher education (199,433; 37.7%). The study population was mostly aged between 35 and 54 years of age (175,108; 33.1%).

**Table 2:** Cohort characteristics

Characteristics	Original Cohort	Matched Sample*		
		Controls	Used Healthcare due to Violence	Total
Sex				
Male	1,386,067 (44.0%)	151,481 (28.7%)	651 (31.9%)	152,132 (28.7%)
Female	1,762,316 (56.0%)	375,700 (71.3%)	1,387 (68.1%)	377,087 (71.3%)
Race/Skin Colour				
White	1,085,190 (34.5%)	156,635 (29.7%)	587 (28.8%)	157,222 (29.7%)
Black	365,452 (11.6%)	42,511 (8.1%)	364 (17.9%)	42,875 (8.1%)
Pardo (Mixed Race)	1,574,381 (50.0%)	323,545 (61.4%)	1,038 (50.9%)	324,583 (61.3%)
Amarelo (Asian), Indigenous, Other or Unknown (Missing)	123,360 (3.9%)	4,490 (0.9%)	49 (2.4%)	4,539 (0.9%)
Education				
None/pre-school/literacy class	1,296,861 (41.2%)	47,085 (8.9%)	274 (13.4%)	47,359 (8.9%)
Elementary school	789,131 (25.1%)	215,774 (40.9%)	959 (47.1%)	216,733 (41.0%)
High school or higher education	633,051 (20.1%)	198,900 (37.7%)	533 (26.2%)	199,433 (37.7%)
None reported (missing)	429,340 (13.6%)	65,422 (12.4%)	272 (13.3%)	65,694 (12.4%)
Age Group (years)				
0–18	762,075 (24.2%)	76,794 (14.6%)	240 (11.8%)	77,034 (14.6%)
19–34	768,211 (24.4%)	140,193 (26.6%)	615 (30.2%)	140,808 (26.6%)
35–54	855,136 (27.2%)	174,378 (33.1%)	730 (35.8%)	175,108 (33.1%)
55–64	362,962 (11.5%)	80,333 (15.2%)	266 (13.1%)	80,599 (15.2%)
65+	399,999 (12.7%)	55,483 (10.5%)	187 (9.2%)	55,670 (10.5%)
Household Income				
Some salary reported to less than full minimum salary	314,183 (10.0%)	29,517 (5.6%)	322 (15.8%)	29,839 (5.6%)
More than one minimum salary but less than two minimum salaries	634,445 (20.2%)	95,684 (18.2%)	427 (21.0%)	96,111 (18.2%)
Two minimum salaries or more	226,460 (7.2%)	5,613 (1.1%)	91 (4.5%)	5,704 (1.1%)
No salary reported (unknown or missing)	1,973,295 (62.7%)	396,367 (75.2%)	1,198 (58.8%)	397,565 (75.1%)
Registered with Private Health Insurance				
No	2,858,881 (90.8%)	523,641 (99.3%)	1,986 (97.4%)	525,627 (99.3%)
Yes	289,502 (9.2%)	3,540 (0.7%)	52 (2.6%)	3,592 (0.7%)
Bolsa Família-Claiming Family				
No	2,796,963 (88.8%)	498,256 (94.5%)	1,686 (82.7%)	499,942 (94.5%)
Yes	351,420 (11.2%)	28,925 (5.5%)	352 (17.3%)	29,277 (5.5%)
N	**3**,**148**,**383 (100.0%)**	**527**,**181 (99.6%)**	**2**,**038 (0.4%)**	**529**,**219 (100.0%)**

*Cohort used for analysis; one exposed to multiple controls

**Fig. 2:**
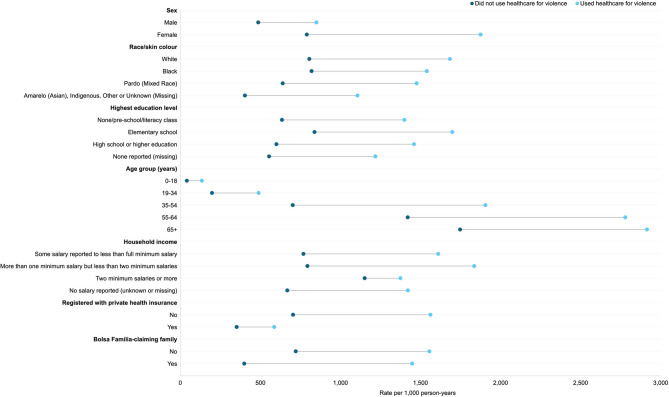
Crude rates of primary healthcare utilisation by sociodemographic variables per 1,000 person-years. See Appendix 6 for full tabulated results

**Fig. 3:**
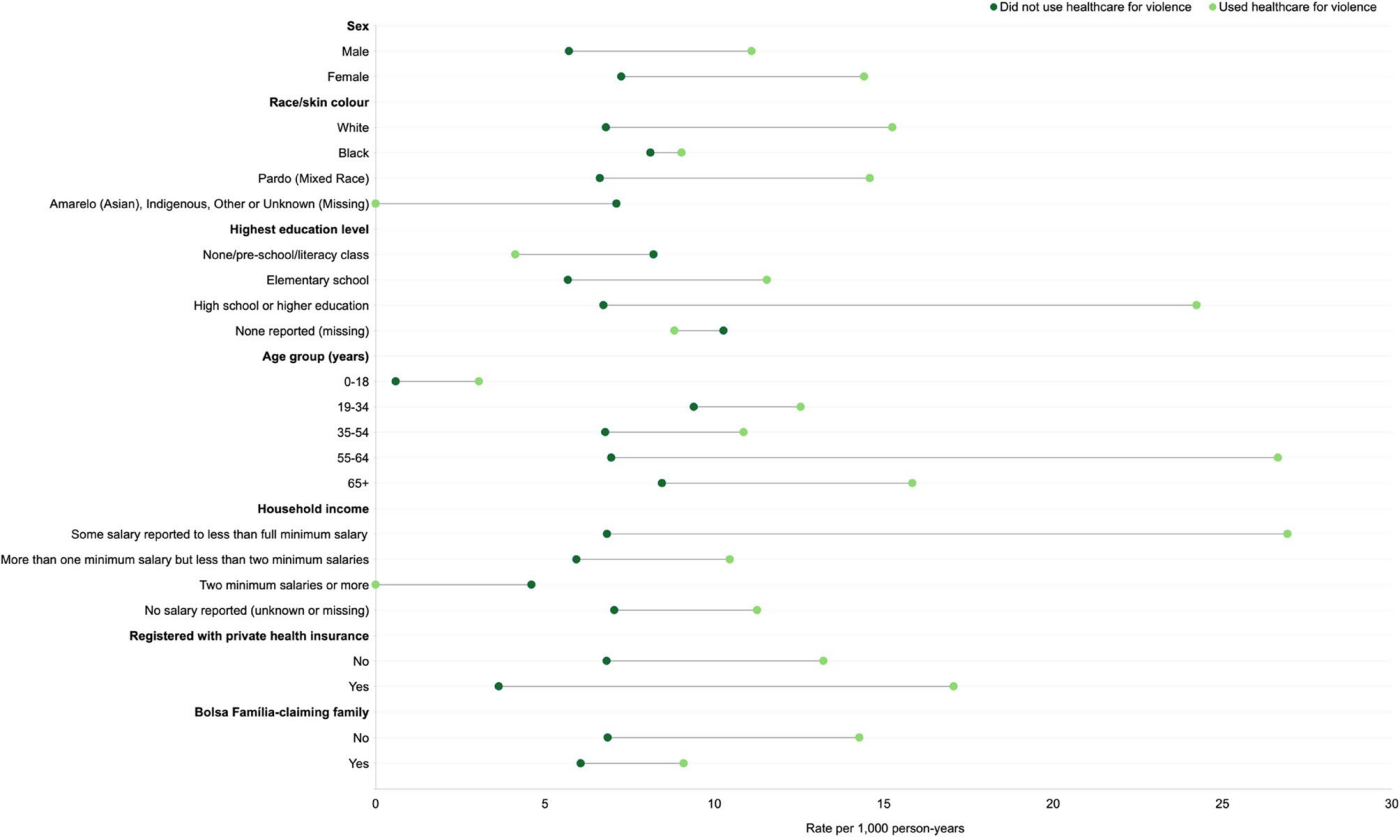
Crude rates of Hospitalisation by sociodemographic variables and whether individuals used healthcare due to violence per 1,000 person-years. See Appendix 6 for full tabulated results

The 2,038 individuals who used healthcare due to violence had a total of 10,029 PHC consultations and 87 Hospital admissions during the study period. Matched controls used PHC 1,024,982 times and were Hospitalised 9,941 times (Appendix 5). Individuals who used healthcare due to violence had over two times the rate of PHC utilisation compared to individuals who did not (1,535.64 compared to 701.23 per 1,000 person-years, respectively) with a similar pattern observed in rates of hospitalisation (13.32 compared to 6.80 per 1,000 person-years, respectively) (Figs. 2 and 3, Appendix 6).

### Patterns of healthcare utilisation over time

In Fixed-effects logistic regression models, individuals who used healthcare due to violence were 11% more likely (OR: 1.11; 95% CI: 1.00–1.24; p<0.05) to use PHC in the first 1-3 months after their initial healthcare contact for violence compared to their period before and to individuals who did not use healthcare due to violence (Table 3). In the longer-term, individuals who used healthcare due to violence were 15% (OR: 0.85; 95% CI: 0.76–0.94; p<0.01) and 22% (OR: 0.78; 95% CI: 0.71–0.85; p<0.005) less likely to use PHC for the selected conditions in the subsequent 7-12 months and >12 months following their initial healthcare contact for violence, respectively. For Hospitalisation, individuals who used healthcare due to violence were 79% less likely (OR: 0.21; 95% CI: 0.05–0.93; p<0.05) to be Hospitalised 7−12 months after their initial healthcare contact for violence compared to individuals who did not use healthcare due to violence. When adjusting for PHC utilisation in the hospitalisation model, individuals who used PHC for the same conditions were more than twice (aOR: 2.08; 95% CI: 1.92–2.26; p<0.005) as likely to be hospitalised for these conditions compared to individuals who did not use PHC for the same conditions (Appendix 7), irrespective of violence exposure.

**Table 3:** Odds ratios from fixed effects logistic regression models of conditions with risk factors associated with using healthcare due to violence for PHC utilisation and hospitalisation by all sociodemographic variables

	PHC Utilisation		Hospitalisation	
Characteristics	OR	95% CI	aOR	95% CI
Months after using healthcare due to violence				
0, or did not use healthcare due to violence	1 (ref)	–	1 (ref)	–
1–3	1.11*	(1.00–1.24)	1.15	(0.48–2.77)
4–6	0.98	(0.87–1.11)	0.78	(0.26–2.35)
7–12	0.85**	(0.76–0.94)	0.21*	(0.05–0.93)
Over 12	0.78***	(0.71–0.85)	0.57	(0.25–1.33)
***Total Observations (N)***	*6*,*802*,*113*		*290*,*393*	
***Total Groups (N)***	*193*,*275*		*7*,*004*	

*Separate fixed effects logistic regressions per outcome (PHC utilisation and hospitalisation), adjusted for year and month*

*PHC Primary Healthcare, OR Odds Ratios, 95% CI 95% Confidence Intervals*

*p<0.05; **p<0.01; ***p<0.001

**Fig. 4:**
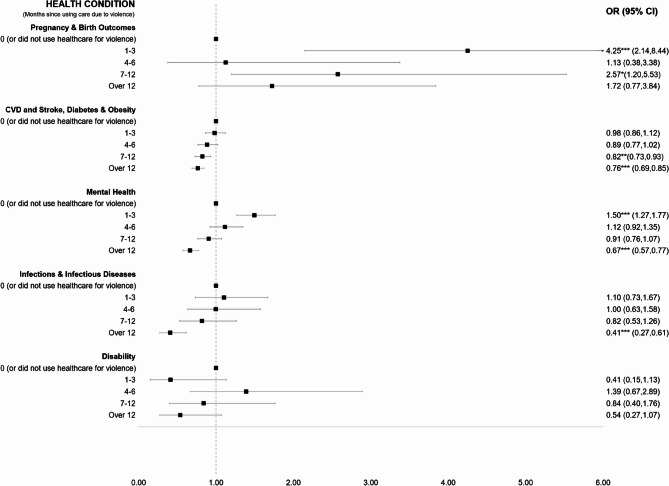
Odds of PHC utilisation months after using care for violence by health condition. Each group of plotted coefficients (per health condition) is from a separate fully adjusted fixed effects logistic regression model. Robust standard errors.*p < 0.05; **p < 0.01; ***p < 0.001. 95% CI – 95% Confidence Intervals. Pregnancy & Birth Outcomes model was restricted to females

In regression analyses by health condition (Figure 4, see Appendix 8 for regression output), females who used healthcare due to violence were 4.25 times more likely (aOR: 4.25; 95% CI: 2.14–8.44; p<0.001) and 2.57 times more likely (aOR: 2.57; 95% CI: 1.20–5.53; p<0.05) to use PHC for poor pregnancy and birth outcomes 1-3 months and 7-12 months after using healthcare due to violence, respectively. Individuals who used healthcare due to violence were 18% (aOR: 0.82; 95% CI: 0.73–0.93; p<0.01) and 24% (aOR: 0.76; 95% CI: 0.69–0.65; p<0.005) less likely to use PHC for cardiovascular disease, diabetes, and obesity 7-12 months and over 12 months after using healthcare due to violence. PHC utilisation for mental health in individuals who used healthcare due to violence increased by 50% (aOR: 1.50; 95% CI: 1.27–1.77; p<0.005) in the first 1-3 months after seeking care due to violence, but decreased by 33% (aOR: 0.67; 95% CI: 0.57–0.77; p<0.005) after 12 months. After 12 months, individuals who used healthcare due to violence were almost 60% (aOR: 0.41; 95% CI: 0.27–0.61; p<0.005) less likely to use PHC for infections and infectious diseases.

Due to low numbers, analyses for the hospitalisation outcome measure were not conducted in the condition-disaggregated subgroups. Additionally, all interactions between key socioeconomic variables (sex, race/skin colour, education level, and household income) and months since using PHC due to violence were not significant in any model specification (Appendices 9, 10, 11, 12).

### Sensitivity analyses

Sensitivity analyses were consistent with the findings from our primary models. Coefficients from the random-effects logistic regression were comparable to the fixed-effects models, indicating no substantial influence of unobserved heterogeneity across individuals (Appendix 13). Similarly, the fixed-effects Poisson regression, which incorporated robust standard errors, yielded estimates that aligned closely with the primary analysis (Appendix 14). Finally, the generalised estimating equations (GEE) approach confirmed the robustness of our results, with population-averaged associations showing no meaningful deviations from the individual-level findings (Appendix 15). Across all alternative model specifications, the direction, magnitude, and significance of associations remained stable, reinforcing the robustness and reliability of our findings.

## Discussion

Low-income individuals in Rio de Janeiro who experienced violence had increased subsequent healthcare use in the short term, notably with increased PHC use in the first six months. This effect was mainly driven by consultations for conditions relating to adverse pregnancy and birth outcomes, mental health, infectious diseases and disability associated outcomes. This effect did not appear to vary across socioeconomic groups, but in the longer-term, individuals who experienced violence had reduced healthcare use, mainly for CVD and stroke, diabetes and obesity, mental health, and infections and infectious diseases.

The finding of a short-term increase in PHC utilisation after experiencing violence is concordant with the wider literature.[8, 19, 20] It may reflect increased healthcare needs due to direct harms or for stress-induced health concerns. The dissipation of this effect over time suggests healthcare needs may have been addressed, and there is some evidence of lower PHC utilisation after 12 months for certain conditions.[55] In contexts of violence, both patients and healthcare providers may deprioritise chronic conditions or long-term care, contributing to a decline in healthcare utilisation over time. This longer-term decline may also be explained by barriers to access, reduced healthcare availability, or shifts in healthcare priorities, as observed in this study.

In this study, PHC utilisation for mental health was one of conditions associated with violence, aligning with evidence that violence exposure is associated with poor mental health [56, 57]. Studies in Brazil and other Latin American countries have demonstrated associations between residing in high-violence areas and risk of having common mental disorders[57–60]. Violence can trigger acute mental health crises such as post-traumatic stress disorder and anxiety, prompting health-seeking. Over time, the association between violence and higher PHC use for mental health may diminish due to symptom relief, increased recovery self-efficacy, or individuals discontinuing care as their symptoms ease. A similar pattern was observed for other conditions, including cardiovascular disease (CVD), diabetes, obesity, and infections. For CVD, relief from chest pain or shortness of breath may lead to fewer PHC visits. In diabetes and obesity, better management of blood sugar levels and weight loss can reduce the need for visits. Similarly, in infections, the resolution of fever or pain often leads to discontinuing treatment[61, 62].

PHC utilisation for pregnancy and birth outcomes rises sharply in the short term after using healthcare due to violence in this study. Violence is strongly associated to adverse pregnancy and birth outcomes in high-income countries and LMICs alike, including Brazil [32, 63–66]. Violence is linked to adverse pregnancy outcomes globally, associated with injuries, stress, and high-risk behaviors (e.g., alcohol, smoking), which contribute to low birth weight and preterm birth [3, 65, 67]. Concern for the unborn or newborn child likely drives increased PHC visits which can lead to poor psychological health and may explain increased PHC use for mental health as well [68]. Similarly, PHC utilisation for infections, infectious diseases, and disability-related outcomes also rises in the months following violence-related care. Evidence shows that violence, notably injury, can lead to increased infection rates [69–71] and disability [72, 73]. Care-seeking in the 4-6 months after seeking care for violence may result from the gradual onset of chronic symptoms like pain and musculoskeletal injuries, and as infections become symptomatic, prompting individuals to seek additional care [70].

Our study found that individuals who used PHC for the selected conditions after experiencing violence, were more than twice as likely to be hospitalised for those same conditions, compared to those who did not use PHC for these conditions. Additionally, we did not find any statistically significant interactions between sex, race/skin colour, education level and income and post-violence PHC utilisation. One plausible explanation is that this study was underpowered to detect inequalities in this association. It may also be the case that increases in PHC use after violence is similar across socioeconomic groups. However, this is unlikely to be the case given well-established differences in exposure to violence, PHC utilisation, and inequalities in violence-related mortality by sex [74–77]. This stems from greater morbidity in women, gender-based social hierarchies, and differing health perceptions both internationally [78–82] and in Brazil [83–85]. Victims of violence are often Black or Pardo, male, and from lower-income or lower-educational backgrounds [75, 76, 86, 87], suggesting further evidence is needed to explore socioeconomic inequalities in the healthcare impacts of violence.

 A key strength of this study was the use of individual-level data with linked health and socioeconomic data facilitating comprehensive analyses. The screening of clinical free text records to identify individuals who experienced violence is another strength, and novel for a study from a LMIC. The CEM achieved multiple exact matches for 2,038 of the 2,049 exposed individuals improving covariate balance, reducing the potential selection bias, and retaining a large proportion of exposed individuals.

Nevertheless, there are limitations. CEM reduced our study population, but sensitivity analyses showed the matched sample was representative of the original 3.1 million cohort. Sociodemographic data was self-reported with potential for misclassification, but is unlikely that biases were sufficiently large to invalidate the findings or correlated with exposure or outcomes. The study population consisted of individuals that used PHC, suggesting the true healthcare impacts of violence are under-reported. As our study population was already using healthcare, increases in utilisation after using healthcare due to violence may seem smaller, potentially underestimating violence’s true effect on healthcare use. Additionally, since women are more likely to use PHC services, they are potentially overrepresented in the sample, reflecting broader healthcare-seeking patterns rather than a specific bias toward certain types of violence. Socioeconomically disadvantaged populations experience greater barriers in access to healthcare, suggesting that underestimations are greater in these populations. Given the low-income focus of the study population, the findings may not be generalisable to the entire city or other settings. Another limitation is that violence exposure was defined by PHC consultations or hospitalisations following a violent event and therefore excludes individuals that were exposed to violence and did not use care and those that may have been exposed to extreme violence which led to death or unreported emergency care. This is likely substantially under-estimating the true healthcare impacts of violence. To mitigate selection bias from defining exposure through healthcare use, we limited our analysis to healthcare users. However, this could introduce bias by potentially overestimating the effects of violence, as those who do not seek care were excluded.

Additionally, we used broad inclusion criteria for healthcare visits related to violence, encompassing any mention of violence regardless of timing. This may reduce specificity in distinguishing recent versus past, or direct versus indirect exposures. Finally, it is possible that some cases of domestic violence including rape and sexual abuse/assault were inadvertently included despite efforts to exclude them, for example by relying on ICD-10 and ICPC codes. Although free-text screening was conducted in an attempt to both identify further cases of violence not coded by ICD-10 and ICPC and to minimise the inclusion of domestic violence cases, complete exclusion is challenging given data limitations.

The findings of this study underscore the need for comprehensive and multifaceted policy interventions to mitigate the adverse health effects associated with violence in Rio de Janeiro. Given the associations between violence and healthcare utilisation were predominantly in the short-term, it is important to strengthen health services to effectively respond to violence-related health issues. Immediate actions could include an increase in mental health and maternal care services in high-risk areas and ensuring efficient referral pathways to specialised services. Enhanced training for healthcare providers in PHC clinics which are often Brazilians’ first point of contact with the health system, especially in low-income areas, is important [88]. Improving access to healthcare and ensuring health facilities are adequately equipped and staffed to handle violence-related cases, whilst ensuring the safety of healthcare workers is essential. Long-term strategies should address the root causes of violence, investing in social and economic development initiatives and public services to disrupt the cycles of poverty, socioeconomic inequality, and consequently violence. Overall, a collaborative approach involving local government, public, and social services, is essential to create a safer and consequently, healthier urban environment.

## Conclusion

There were short-term increases in PHC utilisation in the first six months for individuals using healthcare due to violence in Rio de Janeiro, Brazil. This increase in PHC utilisation is largely driven by a higher number of PHC consultations for poor pregnancy and birth outcomes, poor mental health infections, and disability associated outcomes.

## Data Availability

The datasets generated and analysed during the current study are not publicly available due confidentiality of the linked data. They are available from the corresponding author on reasonable request and following approval from CONEP and CEP.

## Supplemen Information

Supplementary Material 1

## Appendix 1 Development of a conceptual framework of the pathways through which violence increases health risk factors

Applying and expanding Scott-Storey’s [21] and Guha-Sapir & van Panhuis’ [22] frameworks on violence and health, we developed a conceptual framework illustrating four key pathways through which violence in general increases health risk factors (Fig. 1). This conceptual framework was then used to inform the health conditions to consider as PHC and hospitalisation outcomes in the study.

First, chronic exposure to violence triggers neuroendocrine, metabolic, and immunological changes, resulting in chronic inflammation and elevated blood pressure, key markers of cardiovascular risk. Research in Sweden has shown that violence increases coronary heart disease by 75% and 39% in females and males, respectively [88]. Second, coping strategies including smoking, substance abuse, and overeating, adopted in response to stress from violence, persist even after abuse ends, exacerbating cardiovascular risk factors such as high cholesterol, obesity, and diabetes. Evidence from high income countries including Australia and the United States has demonstrated associations between neighbourhood violence and increased body mass index and type 2 diabetes, respectively [31, 89]. Similarly, in Brazil, women living in the least socially cohesive neighbourhoods and those considered the most violent had a 25% increased risk of obesity [90].

Third, violence is strongly associated with poor mental health [91, 92]. Violence’s toll on mental health, specifically depression, contributes to cardiovascular disease through elevated cortisol levels and inflammatory markers. Violence amplifies chronic stress and associated health risks. Elevated cortisol levels from stress, exacerbated by experiencing violence, can disrupt women’s reproductive health, leading to adverse pregnancy outcomes as evidenced in the United States [32]. In low- and middle-income countries, especially in Latin America, violence and poor neighbourhood socioeconomic conditions hampers youth access to sexual and reproductive healthcare, contributing to higher rates of adolescent births [17, 93, 94]. Limited access to healthcare due to violence also heightens the risk of sexually transmitted infections, as seen in cases of intimate partner violence [95, 96]. Lastly, violence-related injuries, including those from weapons, increase infection rates [69–71] and may result in long-term disabilities like paralysis and chronic pain [72, 73].

## Appendix 2 Methods for violence free text search of PHC consultation records

The free text search covered terms (in Portuguese) such as ‘bullet’, ‘violence’, ‘aggression’, ‘assault’, ‘crime’, ‘robbery’, ‘trafficker’, ‘assassinated’ and excluded terms related to domestic violence including ‘domestic’, ‘partner’, ‘husband’, ‘wife’, ‘home’, ‘residence’, ‘suspected’, and ‘negligence’ – to ensure the focus remained on violence outside the home and perpetrated by strangers. Records were included if there was any mention of violence in the consultation record, regardless of the time since occurrence and whether exposure was direct (e.g. victim, injury) or indirect (e.g. loss of a loved one, witnessing urban violence). Spelling errors and typographical errors were accounted for by including both the noun and verb forms of associated terms and using truncations where appropriate.

To validate the search, a random sample of 100 records per violence sub-category (weapon & firearms; physical assault & injury; crime & gang activity) were hand-screened, totalling 300 records, to ensure only relevant cases of violence were identified. Sub-categories of violence were used to facilitate the search and screening process, but were combined for the analysis. If incorrect terms were identified in the screening process, the search was modified, and screening was repeated. Sample screening was repeated until a plateau was reached, defined as minimal changes in precision (true positives/(true positives + false positives)) and the number of records identified by the search, indicating that further screening would not significantly improve results. In total, we performed eight rounds of sample screening. In the final round of screening all 2,734 records identified were manually screened (weapon & firearms, 823; physical assault & injury, 1,443; crime & gang activity, 468).

**Table 4:** Final* violence free text search per violence category

	Search Strategy
Weapon & Firearm	(\bTIRO\b.*|^\bTIRO\b.*|\bTIROTEIO\b|^\bARMA\b.*|\b(ARMA|DO|A)DO\b.*|PISTOL.*|ESFAQUEA.*|FACAD.*|BALEAD.*|FERID.* FACA) AND NOT (NAMORAD.*|PARCEIR.*|MARIDO|MULHER|ESPOS.*|FAM.*|CONJUGE|COMPANHEIR.*|CONSORTE|DOMESTIC.*|CASA|RESIDENCIA|FAMILIA.*|ESTUPR.*|CONJUGAL|NEGLIG.*|SUSPEIT.*|SEXUAL|NOIV.*) AND NOT (TIROID.*|LEVOTIROXINA|TIRO DUVIDAS|REFACA|FARMACIA|RETIROU|DIALEVOTIROXINA|FARMA.*|TUMEFAC.*|FARMACEUTICO|FARMACOLOGICO|ALARMA|TIROU|RETIRO|AFERIDA|RETIRADA|HIPERTIROIDISMO|LECOTIROXINA|ARMARIO|CORRIMENTO|\bDOR TIPO FACAD.*\b|\bCOMO UMA FACADA\b|\bDOR EM FACADA\b|\bTIPO FACAD.*\b|\bEM FACADA\b|\bTIRO A.*\b|\bTIRO G.*\b|\bTIRO D.*\b|\bTIRO O DIAG.*\b|\bCOMO SE FOSSE FACAD.*\b|bCOMO SE FOSSE FACAD.*\b|\bCOMO UMA FACAD.*\b|\bTIRO S.*\b|\bEM FACAD.*\b|ALTERA.*|\bSENSA.* DE FACAD.*\b|\bESTOU MEIO BALEAD.*\b|\bTIRO OS PONTO.*\b|PISTOLA PNEUMATICA|CORRINMENTO|\bEM FACADA.*\b|NAO TIRO ELE|NUNCA TIRO|PISTOLA DE PINO|\bTIRO O DIANG.*\b|GRUPO DE ENTREGA|MATOU A M.*|CORRIEM.*|AUTIAGRESSAO|QUE FACA EXAME|SENSACAO DE FACADAS|COMO SE FOSSE UMA FACADA)
Assault & Injury	(\bTIRO\b.*|^\bTIRO\b.*|\bTIROTEIO\b|^\bARMA\b.*|\b(ARMA|DO|A)DO\b.*|PISTOL.*|ESFAQUEA.*|FACAD.*|BALEAD.*|FERID.* FACA) AND NOT (NAMORAD.*|PARCEIR.*|MARIDO|MULHER|ESPOS.*|FAM.*|CONJUGE|COMPANHEIR.*|CONSORTE|DOMESTIC.*|CASA|RESIDENCIA|FAMILIA.*|ESTUPR.*|CONJUGAL|NEGLIG.*|SUSPEIT.*|SEXUAL|NOIV.*) AND NOT (COMPANHEIR.*\sQUE\sTEM\sQUADROS\sDE\sAGRESSIVIDADE|AGRESSIVA/AGRESSIVO|VIOLETA|AGRESSAO\sFISICA\sDE\sNAMORADO|NEGA\sAGRESSÃO|PACIENTE\sAPRESENTA/COM\sAGRESSIVIDADE|CRIANCA\sESTA\sAGRESSIVA|VIOLENCIA\sSEXUAL|ERITEMATOVIOLACEAS|DOR\sVIOLENTA|AGRESSAO\sFISICA\sFACADA\sPOR\sPARTE\sD.*|APRESENTA-SE\sAGRESSIVA|EVIOLUCAO|AGRESSIVO|AGRESSIVA|VIOLACE.*|RESSALTA|AGRESSIVIDA.*|\bCOR VIOL.*\b|VIOLA.*|VIOLINO|VIOLÃ|AUTO AGRESSAO|AUTO-AGRESSAO|PESSOA CONHECIDA|ESCOLA|COMPORTAMENTO|HETEROAGRESSAO|AGRESSAO POR PARTE|\bAGRESSAO D.*\b|CACHORRO|AGRESSAO SOLAR|EPISODIOS DE AGRESSIV.*|VIOLUMEN|\bPELO SEU COMPANHEIR.*|O FILHO A MALTRATA|EPISODIOS DE AGRESSAO|CRIANCA AGITADA|AGRESS.* INFANTIL|OBSTETRICA|MAGRESSA|AGRESS.* A.* FILH.*|HETEROAGRESS.*|AGRESS.* CONTRA A FILHA|AGRESS.* CONTRA O FILHO.*|NEGA VIOL.*|NEGA AGRESS.*|ELE E VIOL.*|MAE A VIOLENTOU.*|IMPULSOS VIOLENTOS)
Crime & Gang	(\bTIRO\b.*|^\bTIRO\b.*|\bTIROTEIO\b|^\bARMA\b.*|\b(ARMA|DO|A)DO\b.*|PISTOL.*|ESFAQUEA.*|FACAD.*|BALEAD.*|FERID.* FACA) AND NOT (NAMORAD.*|PARCEIR.*|MARIDO|MULHER|ESPOS.*|FAM.*|CONJUGE|COMPANHEIR.*|CONSORTE|DOMESTIC.*|CASA|RESIDENCIA|FAMILIA.*|ESTUPR.*|CONJUGAL|NEGLIG.*|SUSPEIT.*|SEXUAL|NOIV.*) AND NOT (LACRIMEJA|LACRIMEJAMENTO|GANGLIO.*|GANGLIONAR|LACRIMAL|LACRIMEJANDO|LACRIMA|INFACCAO|PARA\sEL.*\sN.*\sROUBAR.*\sAS\sCOISAS|ENDOC.*|LAVCRIME.*|LACRIMEO|GANGRE.*|GANGK.*|GANGIO.*|GANGATE|GANGATA|LACRIME.*|\bASSINAD.*\b|ALECRIM|DISCRIM.*|TUMEFAC.*|GANGO.*|GARGANGTA|GANGANGTA|GANGLOS|GANGLONAR|GANGLI|GANGLIAO|MOSTROUB|HIPOCRIMICA|ACRIMEJAMENTO|BACRIM|LACRIMRJAMENTO|CRIMENTO|LACRIMMASAL|HIPERCRIMICAS|LACRIMIJANDO|LINGANGITES|GANGANTA|GARGANGANTA|GANGILIO.*|RUBEFACCAO|GANGGLIO|GANGRANA|DARACRIM|LACRIMONASAL|REFACCAO|FUROUB|HASSASSIN.*|LECRIMEJ.*|PROVUROUB.*|MASSASSINAL|LCRIMEJAMENT.*|CRIMA|ASSINTOMATICO|ACRIMIO|GANGRIO|GANGARTA|LACRIMOGENEO|FILHA A ROUBA|FILHO A AGRIDE|ASSASSINAR-LA|VOU ROUBAR|LACRIM.*|SENTE IMPULSO DE ROUBAR|IRM.* ROUBOU|JA ROUBOU VARIAS COISAS)

Sources: Nakamura et al. [97] and authors’ own work

*Prior to manual screening of records identified by search

## Appendix 3 International classification of disease (ICD-10) and international classification of primary care (ICPC) codes for analysis of health conditions with risk factors associated with experiencing violence

**Table:**

HEALTH CONDITION (ANALYSIS GROUPS)	ICD-10	CODES	ICPC	CODES
	CATEGORIES		CATEGORIES	
Adverse Pregnancy and Birth Outcomes	Outcome of delivery (including stillbirth)	Z37.1, Z37.3, Z37.4, Z37.6, Z37.7	Bleeding	W03, W17
	Premature rupture of membranes	O42	Pregnancy complications	W27, W29, W71, W75, W80, W81
	Preeclampsia	O11, O14, O15	Post-partum symptom/complaint other	W18
	Gestational hypertension	O13, O16	Unwanted pregnancy	W79
	Gestational diabetes	O24.4	Outcome of delivery	W92, W93, W99, A93
	Poor foetal growth	O36.5, O36.8, O36.9	Abortion	W82, W83
	Placental abruption	O45, O46	Gestational diabetes	W85
	Spontaneous preterm labour	O60.1		
	Other conditions complicating pregnancy	O99		
Cardiovascular Disease & Stroke	Hypertensive diseases	I10-I15	Pain, complaints, and risk factors for cardiovascular disease	K01-K03, K22, K29, A11
	Ischaemic heart diseases	I20-I25	Abnormal heart rhythms	K04, K05, K78-K81
	Heart failure	I50	Ischaemic heart disease	K74-K76, K89-K94
	Cardiac arrest	I46	Heart disease and heart failure	K77, K82-K84, K99
	Cerebrovascular diseases	I60-I69	Hypertensive diseases	K85-K88
	Abnormal heart rhythms	I47-I49		
Diabetes	Type 2 diabetes mellitus	E11-E11.9	Diabetes non-insulin dependent	T90
	Abnormal glucose tolerance test (prediabetes)	R73		
Obesity	Obesity	E66-E66.9	Obesity	T82
	BMI for overweight and obese	Z68.25-Z68.45	Overweight	T83
			Weight gain	T07
			Excessive appetite	T02
Mental Health	Substance abuse/dependence disorders	F10-F19	Substance abuse/dependence disorders	P15, P17-P19
	Psychotic syndromes	F20-F25, F28, F29	Psychotic syndromes	P29, P71-P73, P98, P99
	mood affective disorders	F30-F34, F38, F39	mood affective disorders	P03, P76
	Neurotic, stress-related and somatoform disorders (including anxiety)	F40-F45, F48	Neurotic, stress-related and somatoform disorders (including anxiety)	P01, P02, P74, P79, P82
	Personality and behaviour disorders	F50, F51, F60-F63, F91-F94	Personality and behaviour disorders	P80, P86
	Suicide/associated outcomes	X60-X84, R45.851	Suicide/associated outcomes	P77
Infections & Infectious Diseases	Post-traumatic wound infection, not elsewhere classified	T79.3	Infectious disease other/NOS	A78
	Additional codes (B95-B98) to identify infectious agent: • Streptococcus and staphylococcus as the cause of diseases classified to other chapters • Other specified bacterial agents as the cause of diseases classified to other chapters • Viral agents as the cause of diseases classified to other chapters • Other specified infectious agents as the cause of diseases classified to other chapters	B95-B98	Infections of circulatory system	K70
			Blood infections	B70, B71
			Digestive infections	D70-D73
	Infections with a predominantly sexual mode of transmission	A50-A64	HIV-infection/aids	B90
	Contact with and exposure to infections with a predominantly sexual mode of transmission	Z20.2	Female sexually transmitted infections	X70-X73, X90-X92
	Special screening examination for infections with a predominantly sexual mode of transmission and human immunodeficiency virus [HIV]	Z11.3, Z11.4	Male sexually transmitted infections	Y70-Y76
	Other infections with a predominantly sexual mode of transmission complicating pregnancy, childbirth and the puerperium	O98.3		
Disability	Chronic pain	R52.1, R52.2	Pain general/multiple sites	A01
	Amputation	Y83.5, T05, T13.6, T11.6	Paralyisis/weakness	N18
	Paralysis	T14.4		

Sources: Wonca International Classification Committee [98], World Health Organization [44], Sun et al. [99], de Fatima Marinho de Souza et al. [100], Dugan & Shubrook [101], Suissa et al. [102], Ho et al. [103], Glassgow et al. [104]

## Appendix 4 Coarsened exact matching output

(a) Covariate Balance

Multivariate L1 distance: 0 0.8333353

Univariate imbalance:

**Table:**

	L1	Mean	Min	25%	50%	75%	Max
microarea_code	0.02817	-0.03641	1	0	0	0	-3
sex	0.11968	0.11968	0	0	0	0	0
race_skinc	0.07057	0.03622	0	0	0	0	0
age_group	0.15984	0.35934	0	1	0	0	0
education_highest	0.281	0.43787	0	1	1	0	0
hh_income	0.06818	-0.22771	0	0	0	0	0
insurance	0.06516	-0.06516	0	0	0	0	0
bf	0.06363	0.06363	0	0	0	0	0

(b) Matching Summary

Number of strata: 63,004

Number of matched strata: 1,550

**Table:**

	0	1
All	3146334	2049
Matched	527181	2038
Unmatched	2619153	11

Multivariate L1 distance: 5.309e-14

Univariate imbalance:

**Table:**

	L1	Mean	Min	25%	50%	75%	Max
microarea_code	6.90E-14	1.30E-12	0	0	0	0	0
sex	1.50E-14	-1.80E-13	0	0	0	0	0
race_skinc	2.30E-14	1.10E-12	0	0	0	0	0
age_group	6.10E-14	5.50E-13	0	0	0	0	0
education_highest	7.90E-14	1.70E-13	0	0	0	0	0
hh_income	3.00E-14	1.20E-12	0	0	0	0	0
insurance	1.20E-14	2.10E-15	0	0	0	0	0
bf	1.30E-14	2.40E-14	0	0	0	0	0

## Appendix 5 Summary statistics of PHC utilisation and hospitalisation by sociodemographic variables and violence exposure

**Table:**

Characteristics	Counts of PHC Consultations			Counts of Hospitalisations		
	Did Not Use Healthcare due to Violence	Used Healthcare due to Violence	Total	Did Not Use Healthcare due to Violence	Used Healthcare due to Violence	Total
Sex						
Male	206,646	1,837	208,483	2,426	24	2,450
Female	818,336	8,192	826,528	7,515	63	7,578
Race/skin colour						
White	351,867	3,090	354,957	2,971	28	2,999
Black	99,274	1,875	101,149	983	11	994
Pardo (Mixed Race)	567,777	4,857	572,634	5,880	48	5,928
Amarelo (Asian), Indigenous, Other or Unknown (Missing)	6,064	207	6,271	107	0	107
Highest education level						
None/pre-school/literacy class	73,383	1,357	74,740	949	4	953
Elementary school	535,416	5,296	540,712	3,628	36	3,664
High school or higher education	321,059	2,409	323,468	3,600	40	3,640
None reported (missing)	95,124	967	96,091	1,764	7	1,771
Age group (years)						
0–18	8,258	88	8,346	121	2	123
19–34	75,168	935	76,103	3,576	24	3,600
35–54	346,152	4,559	350,711	3,344	26	3,370
55–64	321,357	2,608	323,965	1,574	25	1,599
65+	274,047	1,839	275,886	1,326	10	1,336
Household income						
Some salary reported to less than full minimum salary	69,741	1,735	71,476	620	29	649
More than one minimum salary but less than two	222,564	2,458	225,022	1,663	14	1,677
Two minimum salaries or more	11,015	287	11,302	44	0	44
No salary reported (unknown or missing)	721,662	5,549	727,211	7,614	44	7,658
Registered with Private Health Insurance						
No	1,021,410	9,926	1,031,336	9,904	84	9,988
Yes	3,572	103	3,675	37	3	40
Bolsa Família-Claiming Family						
No	989,742	8,277	998,019	9,406	76	9,482
Yes	35,240	1,752	36,992	535	11	546
N	1,024,982	10,029	1,035,011	9,941	87	10,028

*PHC* primary healthcare

## Appendix 6 Person-years and crude rates of primary healthcare utilisation and hospitalisation (per 1,000 person-years) overall and by sociodemographic variables and whether individuals used healthcare due to violence

**Table:**

Characteristics	Person-Years			Rates of PHC Utilisation			Rates of Hospitalisation		
	Did Not Use Healthcare due to Violence	Used Healthcare due to Violence	Total	Did Not Use Healthcare due to Violence	Used Healthcare due to Violence	Total	Did Not Use Healthcare due to Violence	Used Healthcare due to Violence	Total
Sex									
Male	425,229.50	2,162.11	427,391.60	485.96	849.63	487.80	5.71	11.10	5.73
Female	1,036,465.00	4,368.73	1,040,833.00	789.55	1,875.15	794.10	7.25	14.42	7.28
Race/Skin Colour									
White	436,959.70	1,835.18	438,794.90	805.26	1,683.76	808.94	6.80	15.26	6.83
Black	121,165.20	1,218.13	122,383.30	819.33	1,539.25	826.49	8.11	9.03	8.12
Pardo (Mixed Race)	888,518.60	3,290.43	891,809.00	639.02	1,476.10	642.10	6.62	14.59	6.65
Amarelo (Asian), Indigenous, Other or Unknown (Missing)	15,050.72	187.10	15,237.82	402.90	1,106.35	411.54	7.11	0.00	7.02
Education									
None/pre-school/literacy class	115,684.30	969.63	116,654.00	634.34	1,399.50	640.70	8.20	4.13	8.17
Elementary school	639,046.00	3,117.25	642,163.20	837.84	1,698.93	842.02	5.68	11.55	5.71
High school or higher education	535,251.50	1,650.27	536,901.80	599.83	1,459.76	602.47	6.73	24.24	6.78
None reported (missing)	171,712.40	793.69	172,506.10	553.97	1,218.36	557.03	10.27	8.82	10.27
Age Group (years)									
0–18	204,788.20	655.50	205,443.70	40.32	134.25	40.62	0.59	3.05	0.60
19–34	380,602.90	1,912.88	382,515.80	197.50	488.79	198.95	9.40	12.55	9.41
35–54	493,147.60	2,393.04	495,540.60	701.92	1,905.11	707.73	6.78	10.86	6.80
55–64	226,310.20	938.45	227,248.60	1,419.98	2,779.05	1,425.60	6.96	26.64	7.04
65+	156,845.40	630.97	157,476.40	1,747.24	2,914.54	1,751.92	8.45	15.85	8.48
Household Income									
Some salary reported to less than full minimum salary	90,770.79	1,077.23	91,848.02	768.32	1,610.61	778.20	6.83	26.92	7.07
More than one minimum salary but less than two minimum salaries	280,508.80	1,339.11	281,847.90	793.43	1,835.55	798.38	5.93	10.45	5.95
Two minimum salaries or more	9,570.12	208.72	9,778.84	1,150.98	1,375.03	1,155.76	4.60	0.00	4.50
No salary reported (unknown or missing)	1,080,845.00	3,905.78	1,084,750.00	667.68	1,420.72	670.40	7.04	11.27	7.06
Registered with Private Health Insurance									
No	1,451,525.00	6,355.04	1,457,880.00	703.68	1,561.91	707.42	6.82	13.22	6.85
Yes	10,168.88	175.80	10,344.68	351.27	585.90	355.26	3.64	17.07	3.87
Bolsa Família-Claiming Family									
No	1,373,346.00	5,321.11	1,378,668.00	720.68	1,555.50	723.90	6.85	14.28	6.88
Yes	88,347.85	1,209.73	89,557.57	398.88	1,448.26	413.05	6.06	9.09	6.10
Total	**1**,**461**,**694.00**	**6**,**530.84**	**1**,**468**,**225.00**	**701.23**	**1**,**535.64**	**704.94**	**6.80**	**13.32**	**6.83**

*PHC* Primary Healthcare

## Appendix 7 Odds ratios from a fixed effects logistic regression model of conditions with risk factors associated with using healthcare due to violence for hospitalisation, adjusted for PHC utilisation for the same conditions

**Table:**

Characteristics	OR	95% CI
Months after violence exposure		
0 months or did not seek healthcare for violence	1 (ref)	–
1–3	1.15	(0.48–2.77)
4–6	0.80	(0.27–2.40)
7–12	0.22*	(0.05–0.94)
Over 12	0.56	(0.24–1.31)
PHC Utilisation for selected conditions		
No	1 (ref)	–
Yes	2.08***	(1.92–2.26)
***Total Observations (N)***	*290*,*393*	
***Total Groups (N)***	*7*,*004*	

*Fixed effects logistic regression, adjusted for year and month*

*OR* Odds Ratios, *95% CI* 95% Confidence Intervals

**p*<0.05; ***p*<0.01; ****p*<0.001

## Appendix 8 Odds ratios from fixed effects logistic regression models of conditions with risk factors associated with using healthcare for violence by months following healthcare use for PHC utilisation by all sociodemographic variables by health condition

**Table:**

Characteristics	Pregnancy & Birth Outcomes^a^		CVD and Stroke, Diabetes & Obesity		Mental Health^b^		Infections & Infectious Diseases		Disability	
	OR	95% CI	OR	95% CI	OR	95% CI	OR	95% CI	OR	95% CI
Months after using healthcare for violence										
0, or did not use healthcare for violence	1 (ref)	–	1 (ref)	–	1 (ref)	–	1 (ref)	–	1 (ref)	–
1–3	4.25	(2.14–8.44)	0.98	(0.86–1.12)	1.50	(1.27–1.77)	1.10	(0.73–1.67)	0.41	(0.15–1.13)
4–6	1.13	(0.38–3.38)	0.89	(0.77–1.02)	1.12	(0.92–1.35)	1.00	(0.63–1.58)	1.39	(0.67–2.89)
7–12	2.57	(1.20–5.53)	0.82	(0.73–0.93)	0.91	(0.76–1.07)	0.82	(0.53–1.26)	0.84	(0.40–1.76)
Over 12	1.72	(0.77–3.84)	0.76	(0.69–0.85)	0.67	(0.57–0.77)	0.41	(0.27–0.61)	0.54	(0.27–1.07)
***Total Observations (N)***	*142*,*196*		*5*,*597*,*694*		*1*,*160*,*452*		*452*,*943*		*349*,*260*	
***Total Groups (N)***	*3*,*758*		*159*,*151*		*32*,*320*		*12*,*837*		*7*,*560*	

*Separate fixed effects logistic regressions; adjusted for year and month*

*PHC* Primary Healthcare, *OR* Odds Ratios, *95% CI* 95% Confidence Intervals, *CVD* cardiovascular disease

**p*<0.05; ***p*<0.01; ****p*<0.001

^a^Pregnancy & Birth Outcomes model was restricted to females

## Appendix 9 Odds ratios from a fixed effects logistic regression model of PHC utilisation for conditions with risk factors associated with using healthcare for violence from interactions between by months following healthcare use and sex

**Table:**

Characteristics	OR	95% CI
Months after using healthcare for violence		
0 months or did not seek healthcare for violence	1 (ref)	–
1–3	1.33*	(1.05–1.69)
4–6	1.02	(0.78–1.34)
7–12	0.78	(0.61–1.00)
Over 12	0.91	(0.75–1.12)
Months after violence exposure × Sex		
1–3 × Female	0.80	(0.61–1.05)
4–6 × Female	0.95	(0.70–1.29)
7–12 × Female	1.10	(0.84–1.45)
Over 12 × Female	0.82	(0.65–1.03)
***Total Observations (N)***	*6*,*802*,*113*	
***Total Groups (N)***	*193*,*275*	

*Fixed effects logistic regression, adjusted for year and month*

*OR* Odds Ratios, *95% CI * 95% Confidence Intervals

**p*<0.05; ***p*<0.01; ****p*<0.001

## Appendix 10 Odds ratios from a fixed effects logistic regression model of PHC utilisation for conditions with risk factors associated with using healthcare for violence from interactions between by months following healthcare use and race/skin colour

**Table:**

Characteristics	OR	95% CI
Months after using healthcare for violence		
0 months or did not seek healthcare for violence	1 (ref)	–
1–3	1.10	(0.90–1.34)
4–6	0.87	(0.69–1.09)
7–12	0.96	(0.79–1.16)
Over 12	0.87	(0.74–1.03)
Months after violence exposure × Race/skin colour		
1–3 × Black	0.97	(0.71–1.32)
1–3 × Pardo (Mixed Race)	1.04	(0.81–1.34)
1–3 × Amarelo (Asian), Indigenous, Other or Unknown (Missing)	1.03	(0.47–2.26)
4–6 × Black	1.24	(0.88–1.75)
4–6 × Pardo (Mixed Race)	1.20	(0.91–1.60)
4–6 × Amarelo (Asian), Indigenous, Other or Unknown (Missing)	0.59	(0.24–1.43)
7–12 × Black	0.86	(0.63–1.16)
7–12 × Pardo (Mixed Race)	0.85	(0.66–1.09)
7–12 × Amarelo (Asian), Indigenous, Other or Unknown (Missing)	0.58	(0.30–1.14)
Over 12 × Black	0.86	(0.66–1.11)
Over 12 × Pardo (Mixed Race)	0.88	(0.72–1.09)
Over 12 × Amarelo (Asian), Indigenous, Other or Unknown (Missing)	0.38**	(0.21–0.69)
***Total Observations (N)***	*6*,*802*,*113*	
***Total Groups (N)***	*193*,*275*	

Fixed effects logistic regression, adjusted for year and month

*OR* Odds Ratios, *95% CI* 95% Confidence Intervals

**p*<0.05; ***p*<0.01. ****p*<0.001

## Appendix 11 Odds ratios from a fixed effects logistic regression model of PHC utilisation for conditions with risk factors associated with using healthcare for violence from interactions between by months following healthcare use and highest education level

**Table:**

Characteristics	OR	95% CI
Months after violence exposure		
0 months or did not seek healthcare for violence	1 (ref)	–
1–3	0.92	(0.67–1.27)
4–6	0.94	(0.66–1.33)
7–12	0.64**	(0.47–0.87)
Over 12	0.61***	(0.48–0.78)
Months after violence exposure × Highest education level		
1–3 × Elementary school	1.22	(0.86–1.73)
1–3 × High school or higher education	1.06	(0.72–1.56)
1–3 × None reported (missing)	1.80**	(1.16–2.79)
4–6 × Elementary school	1.04	(0.71–1.54)
4–6 × High school or higher education	1.05	(0.68–1.62)
4–6 × None reported (missing)	1.10	(0.67–1.82)
7–12 × Elementary school	1.31	(0.94–1.84)
7–12 × High school or higher education	1.38	(0.94–2.01)
7–12 × None reported (missing)	1.71*	(1.11–2.62)
Over 12 × Elementary school	1.22	(0.93–1.60)
Over 12 × High school or higher education	1.47*	(1.08–2.00)
Over 12 × None reported (missing)	1.61*	(1.12–2.32)
***Total Observations (N)***	*6*,*802*,*113*	
***Total Groups (N)***	*193*,*275*	

Fixed effects logistic regression, adjusted for year and month

*OR* Odds Ratios, *95% CI* 95% Confidence Intervals

**p*<0.05; ***p*<0.01; ****p*<0.001

## Appendix 12 Odds ratios from a fixed effects logistic regression model of PHC utilisation for conditions with risk factors associated with using healthcare for violence from interactions between by months following healthcare use and household income

**Table:**

Characteristics	OR	95% CI
Months after violence exposure		
0 months or did not seek healthcare for violence	1 (ref)	–
1–3	0.93	(0.71–1.20)
4–6	1.04	(0.79–1.38)
7–12	0.92	(0.71–1.19)
Over 12	0.79*	(0.64–0.98)
Months after violence exposure × Household income		
1–3 × More than one minimum salary but less than two minimum salaries	1.34	(0.95–1.88)
1–3 × Two minimum salaries or more	1.21	(0.64–2.29)
1–3 × No salary reported (unknown or missing)	1.22	(0.91–1.64)
4–6 × More than one minimum salary but less than two minimum salaries	0.84	(0.57–1.24)
4–6 × Two minimum salaries or more	0.96	(0.44–2.08)
4–6 × No salary reported (unknown or missing)	0.96	(0.70–1.33)
7–12 × More than one minimum salary but less than two minimum salaries	1.12	(0.80–1.57)
7–12 × Two minimum salaries or more	0.71	(0.33–1.52)
7–12 × No salary reported (unknown or missing)	0.84	(0.63–1.13)
Over 12 × More than one minimum salary but less than two minimum salaries	0.99	(0.75–1.32)
Over 12 × Two minimum salaries or more	1.53	(0.74–3.15)
Over 12 × No salary reported (unknown or missing)	0.96	(0.75–1.23)
***Total Observations (N)***	*6*,*802*,*113*	
***Total Groups (N*** ***)***	*193*,*275*	

Fixed effects logistic regression, adjusted for year and month

*OR* Odds Ratios, *95% CI* 95% Confidence Intervals

**p*<0.05; ***p*<0.01; ****p*<0.001

## Appendix 13 Adjusted odds ratios from random effects logistic regression models of conditions with risk factors associated with using healthcare due to violence for PHC utilisation and hospitalisation by all sociodemographic variables

**Table:**

	PHC Utilisation		Hospitalisation	
Characteristics	aOR	95% CI	aOR	95% CI
Months after using healthcare due to violence				
0, or did not use healthcare due to violence	1 (ref)	–	1 (ref)	–
1–3	1.36***	(1.20–1.55)	1.95	(0.85–4.52)
4–6	1.15	(1.00–1.33)	1.33	(0.51–3.45)
7–12	0.97	(0.85–1.11)	0.36	(0.11–1.19)
Over 12	0.87	(0.76–1.01)	0.69	(0.35–1.36)
Sex				
Male	1 (ref)	–	1 (ref)	–
Female	1.34	(1.32–1.37)	1.43	(1.34–1.53)
Race/skin colour				
White	1 (ref)	–	1 (ref)	–
Black	1.32***	(1.29–1.36)	1.15**	(1.04–1.26)
Pardo (Mixed Race)	1.03***	(1.01–1.05)	1.02	(0.96–1.08)
Amarelo (Asian), Indigenous, Other or Unknown (Missing)	0.58***	(0.53–0.64)	0.86	(0.65–1.12)
Highest education level				
None/pre-school/literacy class	1 (ref)	–	1 (ref)	–
Elementary school	1.33***	(1.29–1.38)	0.45***	(0.41–0.50)
High school or higher education	1.05**	(1.02–1.08)	0.42***	(0.38–0.47)
None reported (missing)	0.93***	(0.90–0.97)	0.65***	(0.58–0.74)
Age group (years)				
0–18	1 (ref)	–	1 (ref)	–
19–34	5.47	(5.23–5.72)	20.20	(16.42–24.84)
35–54	30.66***	(29.40–31.97)	10.92	(8.87–13.43)
55–64	101.84***	(97.65–106.21)	10.92***	(8.81–13.54)
65+	156.50***	(150.07–163.21)	16.28***	(13.17–20.11)
Household income				
Some salary reported to less than full minimum salary	1 (ref)	–	1 (ref)	–
More than one minimum salary but less than two	1.03	(1.00–1.07)	0.85*	(0.75–0.96)
Two minimum salaries or more	1.34***	(1.25–1.43)	0.70***	(0.48–1.01)
No salary reported (unknown or missing)	0.90***	(0.87–0.93)	0.92***	(0.82–1.03)
Registered with Private Health Insurance				
No	1 (ref)	–	1 (ref)	–
Yes	0.64***	(0.57–0.70)	0.71	(0.49–1.02)
Bolsa Família-Claiming Family				
No	1 (ref)	–	1 (ref)	–
Yes	0.96*	(0.92–1.00)	1.20**	(1.07–1.35)
***Total Observations (N)***	*17*,*875*,*018*		*17*,*875*,*018*	
***Total Groups (N)***	*529*,*219*		*529*,*219*	

Separate fully adjusted random effects logistic regressions per outcome (PHC utilisation and hospitalisation); adjusted for sex, race/skin colour, education level, age group, household income, private health insurance, Bolsa Família-receiving family, year, and month. Robust standard errors

*PHC* Primary Healthcare, *aOR* Adjusted Odds Ratios, *95% CI* 95% Confidence Intervals

**p*<0.05; ***p*<0.01; ****p*<0.001

## Appendix 14 Adjusted rates ratios from Poisson regression models of conditions with risk factors associated with using healthcare due to violence for PHC utilisation and hospitalisation by all sociodemographic variables

**Table:**

	PHC Utilisation		Hospitalisation	
Characteristics	aRR	95% CI	aOR	95% CI
Months after using healthcare due to violence				
0, or did not use healthcare due to violence	1 (ref)	–	1 (ref)	–
1–3	1.03	(0.95–1.12)	1.14	(0.47–2.79)
4–6	0.95	(0.86–1.04)	0.79	(0.27–2.28)
7–12	0.85***	(0.77–0.93)	0.22*	(0.06–0.85)
Over 12	0.77***	(0.69–0.85)	0.59	(0.29–1.17)
***Total Observations (N)***	*6*,*805*,*893*		*290*,*393*	
***Total Groups (N)***	*194*,*389*		*7.004*	

Separate fully adjusted random effects logistic regressions per outcome (PHC utilisation and hospitalisation); adjusted for sex, race/skin colour, education level, age group, household income, private health insurance, Bolsa Família-receiving family, year, and month. Robust standard errors

*PHC* Primary Healthcare, *aRR* Adjusted Rate Ratios, *95% CI* 95% Confidence Intervals

**p*<0.05; ***p*<0.01; ****p*<0.001

## Appendix 15 Adjusted rates ratios from logit-based generalised estimating equation regression models of conditions with risk factors associated with using healthcare due to violence for PHC utilisation and hospitalisation by all sociodemographic variables

**Table:**

	PHC Utilisation		Hospitalisation	
Characteristics	aOR	95% CI	aOR	95% CI
Months after using healthcare due to violence				
0, or did not use healthcare due to violence	1 (ref)	–	1 (ref)	–
1–3	1.54	(1.39–1.69)	1.63	(0.69–3.85)
4–6	1.33	(1.19–1.49)	1.08	(0.34–3.46)
7–12	1.16	(1.05–1.28)	0.23	(0.03–1.83)
Over 12	1.08*	(1.00–1.17)	0.59	(0.23–1.55)
Sex				
Male	1 (ref)	–	1 (ref)	–
Female	1.27**	(1.26–1.29)	1.25	(1.15–1.37)
Race/skin colour				
White	1 (ref)	–	1 (ref)	–
Black	1.11***	(1.09–1.13)	1.08	(0.94–1.23)
Pardo (Mixed Race)	0.99**	(0.98–1.00)	0.98	(0.90–1.07)
Amarelo (Asian), Indigenous, Other or Unknown (Missing)	0.67***	(0.63–0.72)	0.82	(0.56–1.20)
Highest education level				
None/pre-school/literacy class	1 (ref)	–	1 (ref)	–
Elementary school	1.09***	(1.07–1.11)	0.48***	(0.42–0.55)
High school or higher education	0.93***	(0.91–0.95)	0.46***	(0.40–0.53)
None reported (missing)	0.83***	(0.81–0.85)	0.65***	(0.56–0.76)
Age group (years)				
0–18	1 (ref)	–	1 (ref)	–
19–34	4.58	(4.37–4.79)	16.57	(12.27–22.38)
35–54	15.72***	(15.04–16.43)	9.56	(7.07–12.92)
55–64	31.11***	(29.77–32.52)	10.92***	a
65+	38.26***	(36.61–39.99)	13.45***	(9.89–18.29)
Household income				
Some salary reported to less than full minimum salary	1 (ref)	–	1 (ref)	–
More than one minimum salary but less than two	1.01	(0.99–1.03)	0.88	(0.74–1.04)
Two minimum salaries or more	1.19***	(1.14–1.24)	0.72***	(0.44–1.17)
No salary reported (unknown or missing)	0.91***	(0.89–0.93)	0.95***	(0.82–1.11)
Registered with Private Health Insurance				
No	1 (ref)	–	1 (ref)	–
Yes	0.71***	(0.66–0.77)	0.70	(0.41–1.19)
Bolsa Família-Claiming Family				
No	1 (ref)	–	1 (ref)	–
Yes	1.02	(0.99–1.04)	1.08	(0.91–1.28)
*Total Observations (N)*	*17*,*075*,*018*		*17*,*075*,*018*	
*Total Groups (N)*	*529*,*219*		*529*,*219*	

Separate fully adjusted random effects logistic regressions per outcome (PHC utilisation and hospitalisation); adjusted for sex, race/skin colour, education level, age group, household income, private health insurance, Bolsa Família-receiving family, year, and month. Robust standard errors

*PHC* Primary Healthcare, *aOR* Adjusted Odds Ratios, *95% CI* 95% Confidence Intervals

**p*<0.05; ***p*<0.01; ****p*<0.001
