# Supplementary material for: Healthcare utilisation among low-income individuals affected by violence in Rio de Janeiro, Brazil: a retrospective cohort study
Source: BMC Public Health. 2025 Oct 16;25:3508. doi: 10.1186/s12889-025-24567-2 (PMC12532873; doi:10.1186/s12889-025-24567-2)
Supplement: Supplementary file 1 — Supplementary Material 1 [file 12889_2025_24567_MOESM1_ESM.docx]

Appendix 1 | Development of a conceptual framework of the pathways through which violence increases health risk factors.

Applying and expanding Scott-Storey's^1^ and Guha-Sapir & van Panhuis’^2^ frameworks on violence and health, we developed a conceptual framework illustrating four key pathways through which violence in general increases health risk factors (Figure 1). This conceptual framework was then used to inform the health conditions to consider as PHC and hospitalisation outcomes in the study.

First, chronic exposure to violence triggers neuroendocrine, metabolic, and immunological changes, resulting in chronic inflammation and elevated blood pressure, key markers of cardiovascular risk. Research in Sweden has shown that violence increases coronary heart disease by 75% and 39% in females and males, respectively.^3^ Second, coping strategies including smoking, substance abuse, and overeating, adopted in response to stress from violence, persist even after abuse ends, exacerbating cardiovascular risk factors such as high cholesterol, obesity, and diabetes. Evidence from high income countries including Australia and the United States has demonstrated associations between neighbourhood violence and increased body mass index and type 2 diabetes, respectively.^4,5^ Similarly, in Brazil, women living in the least socially cohesive neighbourhoods and those considered the most violent had a 25% increased risk of obesity.^6^

Third, violence is strongly associated with poor mental health.^7,8^ Violence's toll on mental health, specifically depression, contributes to cardiovascular disease through elevated cortisol levels and inflammatory markers. Violence amplifies chronic stress and associated health risks. Elevated cortisol levels from stress, exacerbated by experiencing violence, can disrupt women's reproductive health, leading to adverse pregnancy outcomes as evidenced in the United States.^9^ In low- and middle-income countries, especially in Latin America, violence and poor neighbourhood socioeconomic conditions hampers youth access to sexual and reproductive healthcare, contributing to higher rates of adolescent births.^10-12^ Limited access to healthcare due to violence also heightens the risk of sexually transmitted infections, as seen in cases of intimate partner violence.^13,14^ Lastly, violence-related injuries, including those from weapons, increase infection rates^15-17^ and may result in long-term disabilities like paralysis and chronic pain.^18,19^

Appendix 2 | Methods for violence free text search of PHC consultation records.

The free text search covered terms (in Portuguese) such as ‘bullet’, ‘violence’, ‘aggression’, ‘assault’, ‘crime’, ‘robbery’, ‘trafficker’, ‘assassinated’ and excluded terms related to domestic violence including ‘domestic’, ‘partner’, ‘husband’, ‘wife’, ‘home’, ‘residence’, ‘suspected’, and ‘negligence’ – to ensure the focus remained on violence outside the home and perpetrated by strangers. Records were included if there was any mention of violence in the consultation record, regardless of the time since occurrence and whether exposure was direct (e.g. victim, injury) or indirect (e.g. loss of a loved one, witnessing urban violence). Spelling errors and typographical errors were accounted for by including both the noun and verb forms of associated terms and using truncations where appropriate.

To validate the search, a random sample of 100 records per violence sub-category (weapon & firearms; physical assault & injury; crime & gang activity) were hand-screened, totalling 300 records, to ensure only relevant cases of violence were identified. Sub-categories of violence were used to facilitate the search and screening process, but were combined for the analysis. If incorrect terms were identified in the screening process, the search was modified, and screening was repeated. Sample screening was repeated until a plateau was reached, defined as minimal changes in precision (true positives/(true positives + false positives)) and the number of records identified by the search, indicating that further screening would not significantly improve results. In total, we performed eight rounds of sample screening. In the final round of screening all 2,734 records identified were manually screened (weapon & firearms, 823; physical assault & injury, 1,443; crime & gang activity, 468).

Table A2 | Final* violence free text search per violence category.

|  | **Search Strategy** |
| --- | --- |
| **Weapon & Firearm** | (\bTIRO\b.*\|^\bTIRO\b.*\|\bTIROTEIO\b\|^\bARMA\b.*\|\b(ARMA\|DO\|A)DO\b.*\|PISTOL.*\|ESFAQUEA.*\|FACAD.*\|BALEAD.*\|FERID.* FACA)  AND NOT  (NAMORAD.*\|PARCEIR.*\|MARIDO\|MULHER\|ESPOS.*\|FAM.*\|CONJUGE\|COMPANHEIR.*\|CONSORTE\|DOMESTIC.*\|CASA\|RESIDENCIA\|FAMILIA.*\|ESTUPR.*\|CONJUGAL\|NEGLIG.*\|SUSPEIT.*\|SEXUAL\|NOIV.*)  AND NOT  (TIROID.*\|LEVOTIROXINA\|TIRO DUVIDAS\|REFACA\|FARMACIA\|RETIROU\|DIALEVOTIROXINA\|FARMA.*\|TUMEFAC.*\|FARMACEUTICO\|FARMACOLOGICO\|ALARMA\|TIROU\|RETIRO\|AFERIDA\|RETIRADA\|HIPERTIROIDISMO\|LECOTIROXINA\|ARMARIO\|CORRIMENTO\|\bDOR TIPO FACAD.*\b\|\bCOMO UMA FACADA\b\|\bDOR EM FACADA\b\|\bTIPO FACAD.*\b\|\bEM FACADA\b\|\bTIRO A.*\b\|\bTIRO G.*\b\|\bTIRO D.*\b\|\bTIRO O DIAG.*\b\|\bCOMO SE FOSSE FACAD.*\b\|bCOMO SE FOSSE FACAD.*\b\|\bCOMO UMA FACAD.*\b\|\bTIRO S.*\b\|\bEM FACAD.*\b\|ALTERA.*\|\bSENSA.* DE FACAD.*\b\|\bESTOU MEIO BALEAD.*\b\|\bTIRO OS PONTO.*\b\|PISTOLA PNEUMATICA\|CORRINMENTO\|\bEM FACADA.*\b\|NAO TIRO ELE\|NUNCA TIRO\|PISTOLA DE PINO\|\bTIRO O DIANG.*\b\|GRUPO DE ENTREGA\|MATOU A M.*\|CORRIEM.*\|AUTIAGRESSAO\|QUE FACA EXAME\|SENSACAO DE FACADAS\|COMO SE FOSSE UMA FACADA) |
| **Assault & Injury** | (\bTIRO\b.*\|^\bTIRO\b.*\|\bTIROTEIO\b\|^\bARMA\b.*\|\b(ARMA\|DO\|A)DO\b.*\|PISTOL.*\|ESFAQUEA.*\|FACAD.*\|BALEAD.*\|FERID.* FACA)  AND NOT  (NAMORAD.*\|PARCEIR.*\|MARIDO\|MULHER\|ESPOS.*\|FAM.*\|CONJUGE\|COMPANHEIR.*\|CONSORTE\|DOMESTIC.*\|CASA\|RESIDENCIA\|FAMILIA.*\|ESTUPR.*\|CONJUGAL\|NEGLIG.*\|SUSPEIT.*\|SEXUAL\|NOIV.*)  AND NOT  (COMPANHEIR.*\sQUE\sTEM\sQUADROS\sDE\sAGRESSIVIDADE\|AGRESSIVA/AGRESSIVO\|VIOLETA\|AGRESSAO\sFISICA\sDE\sNAMORADO\|NEGA\sAGRESSÃO\|PACIENTE\sAPRESENTA/COM\sAGRESSIVIDADE\|CRIANCA\sESTA\sAGRESSIVA\|VIOLENCIA\sSEXUAL\|ERITEMATOVIOLACEAS\|DOR\sVIOLENTA\|AGRESSAO\sFISICA\s\(FACADA\)\sPOR\sPARTE\sD.*\|APRESENTA-SE\sAGRESSIVA\|EVIOLUCAO\|AGRESSIVO\|AGRESSIVA\|VIOLACE.*\|RESSALTA\|AGRESSIVIDA.*\|\bCOR VIOL.*\b\|VIOLA.*\|VIOLINO\|VIOLÃ\|AUTO AGRESSAO\|AUTO-AGRESSAO\|PESSOA CONHECIDA\|ESCOLA\|COMPORTAMENTO\|HETEROAGRESSAO\|AGRESSAO POR PARTE\|\bAGRESSAO D.*\b\|CACHORRO\|AGRESSAO SOLAR\|EPISODIOS DE AGRESSIV.*\|VIOLUMEN\|\bPELO SEU COMPANHEIR.*\|O FILHO A MALTRATA\|EPISODIOS DE AGRESSAO\|CRIANCA AGITADA\|AGRESS.* INFANTIL\|OBSTETRICA\|MAGRESSA\|AGRESS.* A.* FILH.*\|HETEROAGRESS.*\|AGRESS.* CONTRA A FILHA\|AGRESS.* CONTRA O FILHO.*\|NEGA VIOL.*\|NEGA AGRESS.*\|ELE E VIOL.*\|MAE A VIOLENTOU.*\|IMPULSOS VIOLENTOS) |
| **Crime & Gang** | (\bTIRO\b.*\|^\bTIRO\b.*\|\bTIROTEIO\b\|^\bARMA\b.*\|\b(ARMA\|DO\|A)DO\b.*\|PISTOL.*\|ESFAQUEA.*\|FACAD.*\|BALEAD.*\|FERID.* FACA)  AND NOT  (NAMORAD.*\|PARCEIR.*\|MARIDO\|MULHER\|ESPOS.*\|FAM.*\|CONJUGE\|COMPANHEIR.*\|CONSORTE\|DOMESTIC.*\|CASA\|RESIDENCIA\|FAMILIA.*\|ESTUPR.*\|CONJUGAL\|NEGLIG.*\|SUSPEIT.*\|SEXUAL\|NOIV.*)  AND NOT  (LACRIMEJA\|LACRIMEJAMENTO\|GANGLIO.*\|GANGLIONAR\|LACRIMAL\|LACRIMEJANDO\|LACRIMA\|INFACCAO\|PARA\sEL.*\sN.*\sROUBAR.*\sAS\sCOISAS\|ENDOC.*\|LAVCRIME.*\|LACRIMEO\|GANGRE.*\|GANGK.*\|GANGIO.*\|GANGATE\|GANGATA\|LACRIME.*\|\bASSINAD.*\b\|ALECRIM\|DISCRIM.*\|TUMEFAC.*\|GANGO.*\|GARGANGTA\|GANGANGTA\|GANGLOS\|GANGLONAR\|GANGLI\|GANGLIAO\|MOSTROUB\|HIPOCRIMICA\|ACRIMEJAMENTO\|BACRIM\|LACRIMRJAMENTO\|CRIMENTO\|LACRIMMASAL\|HIPERCRIMICAS\|LACRIMIJANDO\|LINGANGITES\|GANGANTA\|GARGANGANTA\|GANGILIO.*\|RUBEFACCAO\|GANGGLIO\|GANGRANA\|DARACRIM\|LACRIMONASAL\|REFACCAO\|FUROUB\|HASSASSIN.*\|LECRIMEJ.*\|PROVUROUB.*\|MASSASSINAL\|LCRIMEJAMENT.*\|CRIMA\|ASSINTOMATICO\|ACRIMIO\|GANGRIO\|GANGARTA\|LACRIMOGENEO\|FILHA A ROUBA\|FILHO A AGRIDE\|ASSASSINAR-LA\|VOU ROUBAR\|LACRIM.*\|SENTE IMPULSO DE ROUBAR\|IRM.* ROUBOU\|JA ROUBOU VARIAS COISAS) |

Sources: Nakamura et al.^20^ and authors’ own work. *Prior to manual screening of records identified by search.

Appendix 3 | International Classification of Disease (ICD-10) and International Classification of Primary Care (ICPC) codes for analysis of health conditions with risk factors associated with experiencing violence.

| **HEALTH CONDITION**  **(ANALYSIS GROUPS)** | **ICD-10** | | **ICPC** | |
| --- | --- | --- | --- | --- |
|  | **CATEGORIES** | **CODES** | **CATEGORIES** | **CODES** |
| **Adverse Pregnancy and Birth Outcomes** | Outcome of delivery (including stillbirth) | Z37.1, Z37.3, Z37.4, Z37.6, Z37.7 | Bleeding | W03, W17 |
|  | Premature rupture of membranes | O42 | Pregnancy complications | W27, W29, W71, W75, W80, W81 |
|  | Preeclampsia | O11, O14, O15 | Post-partum symptom/complaint other | W18 |
|  | Gestational hypertension | O13, O16 | Unwanted pregnancy | W79 |
|  | Gestational diabetes | O24.4 | Outcome of delivery | W92, W93, W99, A93 |
|  | Poor foetal growth | O36.5, O36.8, O36.9 | Abortion | W82, W83 |
|  | Placental abruption | O45, O46 | Gestational diabetes | W85 |
|  | Spontaneous preterm labour | O60.1 |  |  |
|  | Other conditions complicating pregnancy | O99 |  |  |
| **Cardiovascular Disease & Stroke** | Hypertensive diseases | I10-I15 | Pain, complaints, and risk factors for cardiovascular disease | K01-K03, K22, K29, A11 |
|  | Ischaemic heart diseases | I20-I25 | Abnormal heart rhythms | K04, K05, K78-K81 |
|  | Heart failure | I50 | Ischaemic heart disease | K74-K76, K89-K94 |
|  | Cardiac arrest | I46 | Heart disease and heart failure | K77, K82-K84, K99 |
|  | Cerebrovascular diseases | I60-I69 | Hypertensive diseases | K85-K88 |
|  | Abnormal heart rhythms | I47-I49 |  |  |
| **Diabetes** | Type 2 diabetes mellitus | E11-E11.9 | Diabetes non-insulin dependent | T90 |
|  | Abnormal glucose tolerance test (prediabetes) | R73 |  |  |
| **Obesity** | Obesity | E66-E66.9 | Obesity | T82 |
|  | BMI for overweight and obese | Z68.25-Z68.45 | Overweight | T83 |
|  |  |  | Weight gain | T07 |
|  |  |  | Excessive appetite | T02 |
| **Mental Health** | Substance abuse/dependence disorders | F10-F19 | Substance abuse/dependence disorders | P15, P17-P19 |
|  | Psychotic syndromes | F20-F25, F28, F29 | Psychotic syndromes | P29, P71-P73, P98, P99 |
|  | mood affective disorders | F30-F34, F38, F39 | mood affective disorders | P03, P76 |
|  | Neurotic, stress-related and somatoform disorders (including anxiety) | F40-F45, F48 | Neurotic, stress-related and somatoform disorders (including anxiety) | P01, P02, P74, P79, P82 |
|  | Personality and behaviour disorders | F50, F51, F60-F63, F91-F94 | Personality and behaviour disorders | P80, P86 |
|  | Suicide/associated outcomes | X60-X84, R45.851 | Suicide/associated outcomes | P77 |
| **Infections & Infectious Diseases** | Post-traumatic wound infection, not elsewhere classified | T79.3 | Infectious disease other/NOS | A78 |
|  | Additional codes (B95-B98) to identify infectious agent:  • Streptococcus and staphylococcus as the cause of diseases classified to other chapters  • Other specified bacterial agents as the cause of diseases classified to other chapters  • Viral agents as the cause of diseases classified to other chapters  • Other specified infectious agents as the cause of diseases classified to other chapters | B95-B98 | Infections of circulatory system | K70 |
|  |  |  | Blood infections | B70, B71 |
|  |  |  | Digestive infections | D70-D73 |
|  |  |  |  |  |
|  |  |  |  |  |
|  | Infections with a predominantly sexual mode of transmission | A50-A64 | HIV-infection/aids | B90 |
|  | Contact with and exposure to infections with a predominantly sexual mode of transmission | Z20.2 | Female sexually transmitted infections | X70-X73, X90-X92 |
|  | Special screening examination for infections with a predominantly sexual mode of transmission and human immunodeficiency virus [HIV] | Z11.3, Z11.4 | Male sexually transmitted infections | Y70-Y76 |
|  | Other infections with a predominantly sexual mode of transmission complicating pregnancy, childbirth and the puerperium | O98.3 |  |  |
| **Disability** | Chronic pain | R52.1, R52.2 | Pain general/multiple sites | A01 |
|  | Amputation | Y83.5, T05, T13.6, T11.6 | Paralyisis/weakness | N18 |
|  | Paralysis | T14.4 |  |  |

Sources: Wonca International Classification Committee,^21^ World Health Organization,^22^ Sun et al.,^23^ de Fatima Marinho de Souza et al.,^24^ Dugan & Shubrook,^25^ Suissa et al.,^26^ Ho et al.,^27^ Glassgow et al.^28^

Appendix 4 | Coarsened exact matching output.

**(a) Covariate Balance**

Multivariate L1 distance: 0 .8333353

Univariate imbalance:

|  | **L1** | **Mean** | **Min** | **25%** | **50%** | **75%** | **Max** |
| --- | --- | --- | --- | --- | --- | --- | --- |
| microarea_code | 0.02817 | -0.03641 | 1 | 0 | 0 | 0 | -3 |
| sex | 0.11968 | 0.11968 | 0 | 0 | 0 | 0 | 0 |
| race_skinc | 0.07057 | 0.03622 | 0 | 0 | 0 | 0 | 0 |
| age_group | 0.15984 | 0.35934 | 0 | 1 | 0 | 0 | 0 |
| education_highest | 0.281 | 0.43787 | 0 | 1 | 1 | 0 | 0 |
| hh_income | 0.06818 | -0.22771 | 0 | 0 | 0 | 0 | 0 |
| insurance | 0.06516 | -0.06516 | 0 | 0 | 0 | 0 | 0 |
| bf | 0.06363 | 0.06363 | 0 | 0 | 0 | 0 | 0 |

**(b) Matching Summary**

Number of strata: 63,004

Number of matched strata: 1,550

|  | **0** | **1** |
| --- | --- | --- |
| All | 3146334 | 2049 |
| Matched | 527181 | 2038 |
| Unmatched | 2619153 | 11 |

Multivariate L1 distance: 5.309e-14

Univariate imbalance:

|  | **L1** | **Mean** | **Min** | **25%** | **50%** | **75%** | **Max** |
| --- | --- | --- | --- | --- | --- | --- | --- |
| microarea_code | 6.90E-14 | 1.30E-12 | 0 | 0 | 0 | 0 | 0 |
| sex | 1.50E-14 | -1.80E-13 | 0 | 0 | 0 | 0 | 0 |
| race_skinc | 2.30E-14 | 1.10E-12 | 0 | 0 | 0 | 0 | 0 |
| age_group | 6.10E-14 | 5.50E-13 | 0 | 0 | 0 | 0 | 0 |
| education_highest | 7.90E-14 | 1.70E-13 | 0 | 0 | 0 | 0 | 0 |
| hh_income | 3.00E-14 | 1.20E-12 | 0 | 0 | 0 | 0 | 0 |
| insurance | 1.20E-14 | 2.10E-15 | 0 | 0 | 0 | 0 | 0 |
| bf | 1.30E-14 | 2.40E-14 | 0 | 0 | 0 | 0 | 0 |

Appendix 5 | Summary statistics of PHC utilisation and hospitalisation by sociodemographic variables and violence exposure.

| **Characteristics** | **Counts of PHC Consultations** | | | **Counts of Hospitalisations** | | |
| --- | --- | --- | --- | --- | --- | --- |
|  | **Did Not Use Healthcare due to Violence** | **Used Healthcare due to Violence** | **Total** | **Did Not Use Healthcare due to Violence** | **Used Healthcare due to Violence** | **Total** |
| **Sex** | | | | | | |
| Male | 206,646 | 1,837 | 208,483 | 2,426 | 24 | 2,450 |
| Female | 818,336 | 8,192 | 826,528 | 7,515 | 63 | 7,578 |
| **Race/skin colour** | | | | | | |
| White | 351,867 | 3,090 | 354,957 | 2,971 | 28 | 2,999 |
| Black | 99,274 | 1,875 | 101,149 | 983 | 11 | 994 |
| Pardo (Mixed Race) | 567,777 | 4,857 | 572,634 | 5,880 | 48 | 5,928 |
| Amarelo (Asian), Indigenous, Other or Unknown (Missing) | 6,064 | 207 | 6,271 | 107 | 0 | 107 |
| **Highest education level** | | | | | | |
| None/pre-school/literacy class | 73,383 | 1,357 | 74,740 | 949 | 4 | 953 |
| Elementary school | 535,416 | 5,296 | 540,712 | 3,628 | 36 | 3,664 |
| High school or higher education | 321,059 | 2,409 | 323,468 | 3,600 | 40 | 3,640 |
| None reported (missing) | 95,124 | 967 | 96,091 | 1,764 | 7 | 1,771 |
| **Age group (years)** | | | | | | |
| 0-18 | 8,258 | 88 | 8,346 | 121 | 2 | 123 |
| 19-34 | 75,168 | 935 | 76,103 | 3,576 | 24 | 3,600 |
| 35-54 | 346,152 | 4,559 | 350,711 | 3,344 | 26 | 3,370 |
| 55-64 | 321,357 | 2,608 | 323,965 | 1,574 | 25 | 1,599 |
| 65+ | 274,047 | 1,839 | 275,886 | 1,326 | 10 | 1,336 |
| **Household income** | | | | | | |
| Some salary reported to less than full minimum salary | 69,741 | 1,735 | 71,476 | 620 | 29 | 649 |
| More than one minimum salary but less than two | 222,564 | 2,458 | 225,022 | 1,663 | 14 | 1,677 |
| Two minimum salaries or more | 11,015 | 287 | 11,302 | 44 | 0 | 44 |
| No salary reported (unknown or missing) | 721,662 | 5,549 | 727,211 | 7,614 | 44 | 7,658 |
| **Registered with Private Health Insurance** | | | | | | |
| No | 1,021,410 | 9,926 | 1,031,336 | 9,904 | 84 | 9,988 |
| Yes | 3,572 | 103 | 3,675 | 37 | 3 | 40 |
| **Bolsa Família-Claiming Family** | | | | | | |
| No | 989,742 | 8,277 | 998,019 | 9,406 | 76 | 9,482 |
| Yes | 35,240 | 1,752 | 36,992 | 535 | 11 | 546 |
| **N** | 1,024,982 | 10,029 | 1,035,011 | 9,941 | 87 | 10,028 |

*PHC – primary healthcare.*

Appendix 6 | Person-years and crude rates of primary healthcare utilisation and hospitalisation (per 1,000 person-years) overall and by sociodemographic variables and whether individuals used healthcare due to violence.

| **Characteristics** | **Person-Years** | | | **Rates of PHC Utilisation** | | | **Rates of Hospitalisation** | | |
| --- | --- | --- | --- | --- | --- | --- | --- | --- | --- |
|  | **Did Not Use Healthcare due to Violence** | **Used Healthcare due to Violence** | **Total** | **Did Not Use Healthcare due to Violence** | **Used Healthcare due to Violence** | **Total** | **Did Not Use Healthcare due to Violence** | **Used Healthcare due to Violence** | **Total** |
| **Sex** | | | | | | | | | |
| Male | 425,229.50 | 2,162.11 | 427,391.60 | 485.96 | 849.63 | 487.80 | 5.71 | 11.10 | 5.73 |
| Female | 1,036,465.00 | 4,368.73 | 1,040,833.00 | 789.55 | 1,875.15 | 794.10 | 7.25 | 14.42 | 7.28 |
| **Race/Skin Colour** | | | | | | | | | |
| White | 436,959.70 | 1,835.18 | 438,794.90 | 805.26 | 1,683.76 | 808.94 | 6.80 | 15.26 | 6.83 |
| Black | 121,165.20 | 1,218.13 | 122,383.30 | 819.33 | 1,539.25 | 826.49 | 8.11 | 9.03 | 8.12 |
| Pardo (Mixed Race) | 888,518.60 | 3,290.43 | 891,809.00 | 639.02 | 1,476.10 | 642.10 | 6.62 | 14.59 | 6.65 |
| Amarelo (Asian), Indigenous, Other or Unknown (Missing) | 15,050.72 | 187.10 | 15,237.82 | 402.90 | 1,106.35 | 411.54 | 7.11 | 0.00 | 7.02 |
| **Education** | | | | | | | | | |
| None/pre-school/literacy class | 115,684.30 | 969.63 | 116,654.00 | 634.34 | 1,399.50 | 640.70 | 8.20 | 4.13 | 8.17 |
| Elementary school | 639,046.00 | 3,117.25 | 642,163.20 | 837.84 | 1,698.93 | 842.02 | 5.68 | 11.55 | 5.71 |
| High school or higher education | 535,251.50 | 1,650.27 | 536,901.80 | 599.83 | 1,459.76 | 602.47 | 6.73 | 24.24 | 6.78 |
| None reported (missing) | 171,712.40 | 793.69 | 172,506.10 | 553.97 | 1,218.36 | 557.03 | 10.27 | 8.82 | 10.27 |
| **Age Group (years)** | | | | | | | | | |
| 0-18 | 204,788.20 | 655.50 | 205,443.70 | 40.32 | 134.25 | 40.62 | 0.59 | 3.05 | 0.60 |
| 19-34 | 380,602.90 | 1,912.88 | 382,515.80 | 197.50 | 488.79 | 198.95 | 9.40 | 12.55 | 9.41 |
| 35-54 | 493,147.60 | 2,393.04 | 495,540.60 | 701.92 | 1,905.11 | 707.73 | 6.78 | 10.86 | 6.80 |
| 55-64 | 226,310.20 | 938.45 | 227,248.60 | 1,419.98 | 2,779.05 | 1,425.60 | 6.96 | 26.64 | 7.04 |
| 65+ | 156,845.40 | 630.97 | 157,476.40 | 1,747.24 | 2,914.54 | 1,751.92 | 8.45 | 15.85 | 8.48 |
| **Household Income** | | | | | | | | | |
| Some salary reported to less than full minimum salary | 90,770.79 | 1,077.23 | 91,848.02 | 768.32 | 1,610.61 | 778.20 | 6.83 | 26.92 | 7.07 |
| More than one minimum salary but less than two minimum salaries | 280,508.80 | 1,339.11 | 281,847.90 | 793.43 | 1,835.55 | 798.38 | 5.93 | 10.45 | 5.95 |
| Two minimum salaries or more | 9,570.12 | 208.72 | 9,778.84 | 1,150.98 | 1,375.03 | 1,155.76 | 4.60 | 0.00 | 4.50 |
| No salary reported (unknown or missing) | 1,080,845.00 | 3,905.78 | 1,084,750.00 | 667.68 | 1,420.72 | 670.40 | 7.04 | 11.27 | 7.06 |
| **Registered with Private Health Insurance** | | | | | | | | | |
| No | 1,451,525.00 | 6,355.04 | 1,457,880.00 | 703.68 | 1,561.91 | 707.42 | 6.82 | 13.22 | 6.85 |
| Yes | 10,168.88 | 175.80 | 10,344.68 | 351.27 | 585.90 | 355.26 | 3.64 | 17.07 | 3.87 |
| **Bolsa Família-Claiming Family** | | | | | | | | | |
| No | 1,373,346.00 | 5,321.11 | 1,378,668.00 | 720.68 | 1,555.50 | 723.90 | 6.85 | 14.28 | 6.88 |
| Yes | 88,347.85 | 1,209.73 | 89,557.57 | 398.88 | 1,448.26 | 413.05 | 6.06 | 9.09 | 6.10 |
| **Total** | **1,461,694.00** | **6,530.84** | **1,468,225.00** | **701.23** | **1,535.64** | **704.94** | **6.80** | **13.32** | **6.83** |

*PHC – Primary Healthcar*

Appendix 7 | Odds ratios from a fixed effects logistic regression model of conditions with risk factors associated with using healthcare due to violence for hospitalisation, adjusted for PHC utilisation for the same conditions.

| **Characteristics** | **OR** |  | **95% CI** |
| --- | --- | --- | --- |
| **Months after violence exposure** |  |  |  |
| 0 months or did not seek healthcare for violence | 1 (ref) |  | – |
| 1-3 | 1.15 |  | (0.48–2.77) |
| 4-6 | 0.80 |  | (0.27–2.40) |
| 7-12 | 0.22 | * | (0.05–0.94) |
| Over 12 | 0.56 |  | (0.24–1.31) |
| **PHC Utilisation for selected conditions** |  |  |  |
| No | 1 (ref) |  | – |
| Yes | 2.08 | *** | (1.92–2.26) |
| ***Total Observations (N)*** | *290,393* | | |
| ***Total Groups (N)*** | *7,004* | | |

*OR – Odds Ratios; 95% CI – 95% Confidence Intervals. Fixed effects logistic regression, adjusted for year and month. *p<0.05; **p<0.01; *** p<0.001.*

Appendix 8 | Odds ratios from fixed effects logistic regression models of conditions with risk factors associated with using healthcare for violence by months following healthcare use for PHC utilisation by all sociodemographic variables by health condition.

|  | **Pregnancy & Birth Outcomes^a^** | | | **CVD and Stroke, Diabetes & Obesity** | | | **Mental Health^b^** | | | **Infections & Infectious Diseases** | | | **Disability** | | |
| --- | --- | --- | --- | --- | --- | --- | --- | --- | --- | --- | --- | --- | --- | --- | --- |
| **Characteristics** | **OR** |  | **95% CI** | **OR** |  | **95% CI** | **OR** |  | **95% CI** | **OR** |  | **95% CI** | **OR** |  | **95% CI** |
| **Months after using healthcare for violence** |  |  |  |  |  |  |  |  |  |  |  |  |  |  |  |
| 0, or did not use healthcare for violence | 1 (ref) |  | – | 1 (ref) |  | – | 1 (ref) |  | – | 1 (ref) |  | – | 1 (ref) |  | – |
| 1-3 | 4.25 | *** | (2.14–8.44) | 0.98 |  | (0.86–1.12) | 1.50 | *** | (1.27–1.77) | 1.10 |  | (0.73–1.67) | 0.41 |  | (0.15–1.13) |
| 4-6 | 1.13 |  | (0.38–3.38) | 0.89 |  | (0.77–1.02) | 1.12 |  | (0.92–1.35) | 1.00 |  | (0.63–1.58) | 1.39 |  | (0.67–2.89) |
| 7-12 | 2.57 | * | (1.20–5.53) | 0.82 | ** | (0.73–0.93) | 0.91 |  | (0.76–1.07) | 0.82 |  | (0.53–1.26) | 0.84 |  | (0.40–1.76) |
| Over 12 | 1.72 |  | (0.77–3.84) | 0.76 | *** | (0.69–0.85) | 0.67 | *** | (0.57–0.77) | 0.41 | *** | (0.27–0.61) | 0.54 |  | (0.27–1.07) |
| ***Total Observations (N)*** | *142,196* | | | *5,597,694* | | | *1,160,452* | | | *452,943* | | | *349,260* | | |
| ***Total Groups (N)*** | *3,758* | | | *159,151* | | | *32,320* | | | *12,837* | | | *7,560* | | |

*PHC – Primary Healthcare;* *OR – Odds Ratios; 95% CI – 95% Confidence Intervals; CVD – cardiovascular disease.*

*Separate fixed effects logistic regressions; adjusted for year and month. *p<0.05; **p<0.01; *** p<0.001.*

*^a^Pregnancy & Birth Outcomes model was restricted to females.*

Appendix 9 | Odds ratios from a fixed effects logistic regression model of PHC utilisation for conditions with risk factors associated with using healthcare for violence from interactions between by months following healthcare use and sex.

| **Characteristics** | **OR** |  | **95% CI** |
| --- | --- | --- | --- |
| **Months after using healthcare for violence** |  |  |  |
| 0 months or did not seek healthcare for violence | 1 (ref) |  | – |
| 1-3 | 1.33 | * | (1.05–1.69) |
| 4-6 | 1.02 |  | (0.78–1.34) |
| 7-12 | 0.78 |  | (0.61–1.00) |
| Over 12 | 0.91 |  | (0.75–1.12) |
| **Months after violence exposure × Sex** |  |  |  |
| 1-3 × Female | 0.80 |  | (0.61–1.05) |
| 4-6 × Female | 0.95 |  | (0.70–1.29) |
| 7-12 × Female | 1.10 |  | (0.84–1.45) |
| Over 12 × Female | 0.82 |  | (0.65–1.03) |
| ***Total Observations (N)*** | *6,802,113* | | |
| ***Total Groups (N)*** | *193,275* | | |

*OR – Odds Ratios; 95% CI – 95% Confidence Intervals. Fixed effects logistic regression, adjusted for year and month. *p<0.05; **p<0.01; *** p<0.001.*

Appendix 10 | Odds ratios from a fixed effects logistic regression model of PHC utilisation for conditions with risk factors associated with using healthcare for violence from interactions between by months following healthcare use and race/skin colour.

| **Characteristics** | **OR** |  | **95% CI** |
| --- | --- | --- | --- |
| **Months after using healthcare for violence** |  |  |  |
| 0 months or did not seek healthcare for violence | 1 (ref) |  | – |
| 1-3 | 1.10 |  | (0.90–1.34) |
| 4-6 | 0.87 |  | (0.69–1.09) |
| 7-12 | 0.96 |  | (0.79–1.16) |
| Over 12 | 0.87 |  | (0.74–1.03) |
| **Months after violence exposure × Race/skin colour** |  |  |  |
| 1-3 × Black | 0.97 |  | (0.71–1.32) |
| 1-3 × Pardo (Mixed Race) | 1.04 |  | (0.81–1.34) |
| 1-3 × Amarelo (Asian), Indigenous, Other or Unknown (Missing) | 1.03 |  | (0.47–2.26) |
| 4-6 × Black | 1.24 |  | (0.88–1.75) |
| 4-6 × Pardo (Mixed Race) | 1.20 |  | (0.91–1.60) |
| 4-6 × Amarelo (Asian), Indigenous, Other or Unknown (Missing) | 0.59 |  | (0.24–1.43) |
| 7-12 × Black | 0.86 |  | (0.63–1.16) |
| 7-12 × Pardo (Mixed Race) | 0.85 |  | (0.66–1.09) |
| 7-12 × Amarelo (Asian), Indigenous, Other or Unknown (Missing) | 0.58 |  | (0.30–1.14) |
| Over 12 × Black | 0.86 |  | (0.66–1.11) |
| Over 12 × Pardo (Mixed Race) | 0.88 |  | (0.72–1.09) |
| Over 12 × Amarelo (Asian), Indigenous, Other or Unknown (Missing) | 0.38 | ** | (0.21–0.69) |
| ***Total Observations (N)*** | *6,802,113* | | |
| ***Total Groups (N)*** | *193,275* | | |

*OR – Odds Ratios; 95% CI – 95% Confidence Intervals. Fixed effects logistic regression, adjusted for year and month. *p<0.05; **p<0.01; *** p<0.001.*

Appendix 11 | Odds ratios from a fixed effects logistic regression model of PHC utilisation for conditions with risk factors associated with using healthcare for violence from interactions between by months following healthcare use and highest education level.

| **Characteristics** | **OR** |  | **95% CI** |
| --- | --- | --- | --- |
| **Months after violence exposure** |  |  |  |
| 0 months or did not seek healthcare for violence | 1 (ref) |  | – |
| 1-3 | 0.92 |  | (0.67–1.27) |
| 4-6 | 0.94 |  | (0.66–1.33) |
| 7-12 | 0.64 | ** | (0.47–0.87) |
| Over 12 | 0.61 | *** | (0.48–0.78) |
| **Months after violence exposure × Highest education level** |  |  |  |
| 1-3 × Elementary school | 1.22 |  | (0.86–1.73) |
| 1-3 × High school or higher education | 1.06 |  | (0.72–1.56) |
| 1-3 × None reported (missing) | 1.80 | ** | (1.16–2.79) |
| 4-6 × Elementary school | 1.04 |  | (0.71–1.54) |
| 4-6 × High school or higher education | 1.05 |  | (0.68–1.62) |
| 4-6 × None reported (missing) | 1.10 |  | (0.67–1.82) |
| 7-12 × Elementary school | 1.31 |  | (0.94–1.84) |
| 7-12 × High school or higher education | 1.38 |  | (0.94–2.01) |
| 7-12 × None reported (missing) | 1.71 | * | (1.11–2.62) |
| Over 12 × Elementary school | 1.22 |  | (0.93–1.60) |
| Over 12 × High school or higher education | 1.47 | * | (1.08–2.00) |
| Over 12 × None reported (missing) | 1.61 | * | (1.12–2.32) |
| ***Total Observations (N)*** | *6,802,113* | | |
| ***Total Groups (N)*** | *193,275* | | |

*OR – Odds Ratios; 95% CI – 95% Confidence Intervals. Fixed effects logistic regression, adjusted for year and month. *p<0.05; **p<0.01; *** p<0.001.*

Appendix 12 | Odds ratios from a fixed effects logistic regression model of PHC utilisation for conditions with risk factors associated with using healthcare for violence from interactions between by months following healthcare use and household income.

| **Characteristics** | **OR** |  | **95% CI** |
| --- | --- | --- | --- |
| **Months after violence exposure** |  |  |  |
| 0 months or did not seek healthcare for violence | 1 (ref) |  | – |
| 1-3 | 0.93 |  | (0.71–1.20) |
| 4-6 | 1.04 |  | (0.79–1.38) |
| 7-12 | 0.92 |  | (0.71–1.19) |
| Over 12 | 0.79 | * | (0.64–0.98) |
| **Months after violence exposure × Household income** |  |  |  |
| 1-3 × More than one minimum salary but less than two minimum salaries | 1.34 |  | (0.95–1.88) |
| 1-3 × Two minimum salaries or more | 1.21 |  | (0.64–2.29) |
| 1-3 × No salary reported (unknown or missing) | 1.22 |  | (0.91–1.64) |
| 4-6 × More than one minimum salary but less than two minimum salaries | 0.84 |  | (0.57–1.24) |
| 4-6 × Two minimum salaries or more | 0.96 |  | (0.44–2.08) |
| 4-6 × No salary reported (unknown or missing) | 0.96 |  | (0.70–1.33) |
| 7-12 × More than one minimum salary but less than two minimum salaries | 1.12 |  | (0.80–1.57) |
| 7-12 × Two minimum salaries or more | 0.71 |  | (0.33–1.52) |
| 7-12 × No salary reported (unknown or missing) | 0.84 |  | (0.63–1.13) |
| Over 12 × More than one minimum salary but less than two minimum salaries | 0.99 |  | (0.75–1.32) |
| Over 12 × Two minimum salaries or more | 1.53 |  | (0.74–3.15) |
| Over 12 × No salary reported (unknown or missing) | 0.96 |  | (0.75–1.23) |
| ***Total Observations (N)*** | *6,802,113* | | |
| ***Total Groups (N)*** | *193,275* | | |

*OR – Odds Ratios; 95% CI – 95% Confidence Intervals. Fixed effects logistic regression, adjusted for year and month. *p<0.05; **p<0.01; *** p<0.001.*

Appendix 13 | Adjusted odds ratios from random effects logistic regression models of conditions with risk factors associated with using healthcare due to violence for PHC utilisation and hospitalisation by all sociodemographic variables.

|  | **PHC Utilisation** | | | |  | **Hospitalisation** | | | |
| --- | --- | --- | --- | --- | --- | --- | --- | --- | --- |
| **Characteristics** | **aOR** |  |  | **95% CI** |  | **aOR** |  |  | **95% CI** |
| **Months after using healthcare due to violence** |  |  |  |  |  |  |  |  |  |
| 0, or did not use healthcare due to violence | 1 (ref) |  |  | – |  | 1 (ref) |  |  | – |
| 1-3 | 1.36 | *** |  | (1.20–1.55) |  | 1.95 |  |  | (0.85–4.52) |
| 4-6 | 1.15 |  |  | (1.00–1.33) |  | 1.33 |  |  | (0.51–3.45) |
| 7-12 | 0.97 |  |  | (0.85–1.11) |  | 0.36 |  |  | (0.11–1.19) |
| Over 12 | 0.87 |  |  | (0.76–1.01) |  | 0.69 |  |  | (0.35–1.36) |
| **Sex** |  |  |  |  |  |  |  |  |  |
| Male | 1 (ref) |  |  | – |  | 1 (ref) |  |  | – |
| Female | 1.34 |  |  | (1.32–1.37) |  | 1.43 |  |  | (1.34–1.53) |
| **Race/skin colour** |  |  |  |  |  |  |  |  |  |
| White | 1 (ref) |  |  | – |  | 1 (ref) |  |  | – |
| Black | 1.32 | *** |  | (1.29–1.36) |  | 1.15 | ** |  | (1.04–1.26) |
| Pardo (Mixed Race) | 1.03 | *** |  | (1.01–1.05) |  | 1.02 |  |  | (0.96–1.08) |
| Amarelo (Asian), Indigenous, Other or Unknown (Missing) | 0.58 | *** |  | (0.53–0.64) |  | 0.86 |  |  | (0.65–1.12) |
| **Highest education level** |  |  |  |  |  |  |  |  |  |
| None/pre-school/literacy class | 1 (ref) |  |  | – |  | 1 (ref) |  |  | – |
| Elementary school | 1.33 | *** |  | (1.29–1.38) |  | 0.45 | *** |  | (0.41–0.50) |
| High school or higher education | 1.05 | ** |  | (1.02–1.08) |  | 0.42 | *** |  | (0.38–0.47) |
| None reported (missing) | 0.93 | *** |  | (0.90–0.97) |  | 0.65 | *** |  | (0.58–0.74) |
| **Age group (years)** |  |  |  |  |  |  |  |  |  |
| 0-18 | 1 (ref) |  |  | – |  | 1 (ref) |  |  | – |
| 19-34 | 5.47 |  |  | (5.23–5.72) |  | 20.20 |  |  | (16.42–24.84) |
| 35-54 | 30.66 | *** |  | (29.40–31.97) |  | 10.92 |  |  | (8.87–13.43) |
| 55-64 | 101.84 | *** |  | (97.65–106.21) |  | 10.92 | *** |  | (8.81–13.54) |
| 65+ | 156.50 | *** |  | (150.07–163.21) |  | 16.28 | *** |  | (13.17–20.11) |
| **Household income** |  |  |  |  |  |  |  |  |  |
| Some salary reported to less than full minimum salary | 1 (ref) |  |  | – |  | 1 (ref) |  |  | – |
| More than one minimum salary but less than two | 1.03 |  |  | (1.00–1.07) |  | 0.85 | * |  | (0.75–0.96) |
| Two minimum salaries or more | 1.34 | *** |  | (1.25–1.43) |  | 0.70 | *** |  | (0.48–1.01) |
| No salary reported (unknown or missing) | 0.90 | *** |  | (0.87–0.93) |  | 0.92 | *** |  | (0.82–1.03) |
| **Registered with Private Health Insurance** |  |  |  |  |  |  |  |  |  |
| No | 1 (ref) |  |  | – |  | 1 (ref) |  |  | – |
| Yes | 0.64 | *** |  | (0.57–0.70) |  | 0.71 |  |  | (0.49–1.02) |
| **Bolsa Família-Claiming Family** |  |  |  |  |  |  |  |  |  |
| No | 1 (ref) |  |  | – |  | 1 (ref) |  |  | – |
| Yes | 0.96 | * |  | (0.92–1.00) |  | 1.20 | ** |  | (1.07–1.35) |
| ***Total Observations (N)*** | *17,875,018* | |  |  |  | *17,875,018* | |  |  |
| ***Total Groups (N)*** | *529,219* | |  |  |  | *529,219* | |  |  |

*PHC – Primary Healthcare; aOR – Adjusted Odds Ratios; 95% CI – 95% Confidence Intervals.*

*Separate fully adjusted random effects logistic regressions per outcome (PHC utilisation and hospitalisation); adjusted for sex, race/skin colour, education level, age group, household income, private health insurance, Bolsa Família-receiving family**,* *year, and month.*

*Robust standard errors.*p<0.05; **p<0.01; *** p<0.001*

Appendix 14 | Adjusted rates ratios from Poisson regression models of conditions with risk factors associated with using healthcare due to violence for PHC utilisation and hospitalisation by all sociodemographic variables.

|  | **PHC Utilisation** | | | |  | **Hospitalisation** | | | |
| --- | --- | --- | --- | --- | --- | --- | --- | --- | --- |
| **Characteristics** | **aRR** |  |  | **95% CI** |  | **aOR** |  |  | **95% CI** |
| **Months after using healthcare due to violence** |  |  |  |  |  |  |  |  |  |
| 0, or did not use healthcare due to violence | 1 (ref) |  |  | – |  | 1 (ref) |  |  | – |
| 1-3 | 1.03 |  |  | (0.95–1.12) |  | 1.14 |  |  | (0.47–2.79) |
| 4-6 | 0.95 |  |  | (0.86–1.04) |  | 0.79 |  |  | (0.27–2.28) |
| 7-12 | 0.85 | *** |  | (0.77–0.93) |  | 0.22 | * |  | (0.06–0.85) |
| Over 12 | 0.77 | *** |  | (0.69–0.85) |  | 0.59 |  |  | (0.29–1.17) |
| ***Total Observations (N)*** | *6,805,893* | |  |  |  | *290,393* | |  |  |
| ***Total Groups (N)*** | *194,389* | |  |  |  | *7.004* | |  |  |

*PHC – Primary Healthcare; aRR – Adjusted Rate Ratios; 95% CI – 95% Confidence Intervals.*

*Separate fully adjusted random effects logistic regressions per outcome (PHC utilisation and hospitalisation); adjusted for sex, race/skin colour, education level, age group, household income, private health insurance, Bolsa Família-receiving family, year, and month.*

*Robust standard errors.*p<0.05; **p<0.01; *** p<0.001*

Appendix 15 | Adjusted rates ratios from logit-based generalised estimating equation regression models of conditions with risk factors associated with using healthcare due to violence for PHC utilisation and hospitalisation by all sociodemographic variables.

|  | **PHC Utilisation** | | | |  | **Hospitalisation** | | | |
| --- | --- | --- | --- | --- | --- | --- | --- | --- | --- |
| **Characteristics** | **aOR** |  |  | **95% CI** |  | **aOR** |  |  | **95% CI** |
| **Months after using healthcare due to violence** |  |  |  |  |  |  |  |  |  |
| 0, or did not use healthcare due to violence | 1 (ref) |  |  | – |  | 1 (ref) |  |  | – |
| 1-3 | 1.54 | *** |  | (1.39–1.69) |  | 1.63 |  |  | (0.69–3.85) |
| 4-6 | 1.33 | *** |  | (1.19–1.49) |  | 1.08 |  |  | (0.34–3.46) |
| 7-12 | 1.16 | ** |  | (1.05–1.28) |  | 0.23 |  |  | (0.03–1.83) |
| Over 12 | 1.08 | * |  | (1.00–1.17) |  | 0.59 |  |  | (0.23–1.55) |
| **Sex** |  |  |  |  |  |  |  |  |  |
| Male | 1 (ref) |  |  | – |  | 1 (ref) |  |  | – |
| Female | 1.27 | ** |  | (1.26–1.29) |  | 1.25 |  |  | (1.15–1.37) |
| **Race/skin colour** |  |  |  |  |  |  |  |  |  |
| White | 1 (ref) |  |  | – |  | 1 (ref) |  |  | – |
| Black | 1.11 | *** |  | (1.09–1.13) |  | 1.08 |  |  | (0.94–1.23) |
| Pardo (Mixed Race) | 0.99 | ** |  | (0.98–1.00) |  | 0.98 |  |  | (0.90–1.07) |
| Amarelo (Asian), Indigenous, Other or Unknown (Missing) | 0.67 | *** |  | (0.63–0.72) |  | 0.82 |  |  | (0.56–1.20) |
| **Highest education level** |  |  |  |  |  |  |  |  |  |
| None/pre-school/literacy class | 1 (ref) |  |  | – |  | 1 (ref) |  |  | – |
| Elementary school | 1.09 | *** |  | (1.07–1.11) |  | 0.48 | *** |  | (0.42–0.55) |
| High school or higher education | 0.93 | *** |  | (0.91–0.95) |  | 0.46 | *** |  | (0.40–0.53) |
| None reported (missing) | 0.83 | *** |  | (0.81–0.85) |  | 0.65 | *** |  | (0.56–0.76) |
| **Age group (years)** |  |  |  |  |  |  |  |  |  |
| 0-18 | 1 (ref) |  |  | – |  | 1 (ref) |  |  | – |
| 19-34 | 4.58 |  |  | (4.37–4.79) |  | 16.57 |  |  | (12.27–22.38) |
| 35-54 | 15.72 | *** |  | (15.04–16.43) |  | 9.56 |  |  | (7.07–12.92) |
| 55-64 | 31.11 | *** |  | (29.77–32.52) |  | 10.92 | *** |  | a |
| 65+ | 38.26 | *** |  | (36.61–39.99) |  | 13.45 | *** |  | (9.89–18.29) |
| **Household income** |  |  |  |  |  |  |  |  |  |
| Some salary reported to less than full minimum salary | 1 (ref) |  |  | – |  | 1 (ref) |  |  | – |
| More than one minimum salary but less than two | 1.01 |  |  | (0.99–1.03) |  | 0.88 |  |  | (0.74–1.04) |
| Two minimum salaries or more | 1.19 | *** |  | (1.14–1.24) |  | 0.72 | *** |  | (0.44–1.17) |
| No salary reported (unknown or missing) | 0.91 | *** |  | (0.89–0.93) |  | 0.95 | *** |  | (0.82–1.11) |
| **Registered with Private Health Insurance** |  |  |  |  |  |  |  |  |  |
| No | 1 (ref) |  |  | – |  | 1 (ref) |  |  | – |
| Yes | 0.71 | *** |  | (0.66–0.77) |  | 0.70 |  |  | (0.41–1.19) |
| **Bolsa Família-Claiming Family** |  |  |  |  |  |  |  |  |  |
| No | 1 (ref) |  |  | – |  | 1 (ref) |  |  | – |
| Yes | 1.02 |  |  | (0.99–1.04) |  | 1.08 |  |  | (0.91–1.28) |
| ***Total Observations (N)*** | *17,075,018* | |  |  |  | *17,075,018* | |  |  |
| ***Total Groups (N)*** | *529,219* | |  |  |  | *529,219* | |  |  |

*PHC – Primary Healthcare; aOR – Adjusted Odds Ratios; 95% CI – 95% Confidence Intervals.*

*Separate fully adjusted random effects logistic regressions per outcome (PHC utilisation and hospitalisation); adjusted for sex, race/skin colour, education level, age group, household income, private health insurance, Bolsa Família-receiving family, year, and month.*

*Robust standard errors.*p<0.05; **p<0.01; *** p<0.001*

**References**

1. Scott-Storey KA. Abuse as a gendered risk factor for cardiovascular disease: a conceptual model. *Journal of Cardiovascular Nursing.* 2013; 28(6): E1-8.

2. Guha-Sapir D, van Panhuis WG. *Armed conflict and public health: a report on knowledge and knowledge gaps*. CRED Brussels. 2002. Available from: <https://dial.uclouvain.be/downloader/downloader.php?pid=boreal:179723&datastream=PDF_01>

3. Sundquist K, Theobald H, Yang M, Li X, Johansson S-E, Sundquist J. Neighborhood violent crime and unemployment increase the risk of coronary heart disease: A multilevel study in an urban setting. *Social Science & Medicine.* 2006; 62(8): 2061-2071.

4. Christian H, Giles-Corti B, Knuiman M, Timperio A, Foster S. The influence of the built environment, social environment and health behaviors on body mass index. Results from RESIDE. *Preventive Medicine.* 2011; 53(1): 57-60.

5. Hanigan M, Heisler M, Choi H. Relationship between county-level crime and diabetes: Mediating effect of physical inactivity. *Prev Med Rep.* 2020; 20: 101220.

6. Chaparro MP, Pina MF, de Oliveira Cardoso L, Santos SM, Barreto SM, Giatti Gonçalves L, et al. The association between the neighbourhood social environment and obesity in Brazil: a cross-sectional analysis of the ELSA-Brasil study. *BMJ Open.* 2019; 9(9): e026800.

7. Fowler PJ, Tompsett CJ, Braciszewski JM, Jacques-Tiura AJ, Baltes BB. Community violence: a meta-analysis on the effect of exposure and mental health outcomes of children and adolescents. *Development and Psychopathology.* 2009; 21(1): 227-259.

8. Lund C, Brooke-Sumner C, Baingana F, Baron EC, Breuer E, Chandra P, et al. Social determinants of mental disorders and the Sustainable Development Goals: a systematic review of reviews. *The lancet psychiatry.* 2018; 5(4): 357-369.

9. Mayne SL, Pool LR, Grobman WA, Kershaw KN. Associations of neighbourhood crime with adverse pregnancy outcomes among women in Chicago: analysis of electronic health records from 2009 to 2013. *Journal of Epidemiology and Community Health.* 2018; 72(3): 230.

10. Green G, Swartz A, Tembo D, Cooper D, George A, Matzopoulos R, et al. A scoping review of how exposure to urban violence impacts youth access to sexual, reproductive and trauma health care in LMICs. *Global Public Health.* 2022: 1-18.

11. United Nations Population Fund. *State of World Population 2013: Motherhood in Childhood-Facing the Challenge of Adolescent Pregnancy.* UN; 2013.

12. Braverman-Bronstein A, Vidaña-Pérez D, Ortigoza AF, Baldovino-Chiquillo L, Diez-Canseco F, Maslowsky J, et al. Adolescent birth rates and the urban social environment in 363 Latin American cities. *BMJ Glob Health.* 2022; 7(10).

13. World Health Organization. Violence against women and HIV/AIDS: critical intersections—intimate partner violence and HIV/AIDS. *Information Bulletin Series.* 2004; 1: 1-9.

14. Wheeler J, Anfinson K, Valvert D, Lungo S. Is violence associated with increased risk behavior among MSM? Evidence from a population-based survey conducted across nine cities in Central America. *Glob Health Action.* 2014; 7: 24814.

15. Acosta Pedemonte NB, Rocchetti NS, Villalba J, Lerman Tenenbaum D, Settecase CJ, Bagilet DH, et al. Bacillus cereus bacteremia in a patient with an abdominal stab wound. *Revista Argentina de Microbiologia.* 2020; 52(2): 115-117.

16. Bodur H, Onguru P, Akıncı E, Kanyılmaz D, Yetkin A, Ozdemir B, et al. Two cases of acute hepatitis after a serial knife stabbing. *Diagnostic Microbiology and Infectious Disease.* 2012; 74(3): 311-312.

17. Baum GR, Baum JT, Hayward D, MacKay BJ. Gunshot Wounds: Ballistics, Pathology, and Treatment Recommendations, with a Focus on Retained Bullets. *Orthopedic Research and Reviews.* 2022; 14: 293-317.

18. Kuchyn I, Horoshko V. Chronic pain in patients with gunshot wounds. *BMC Anesthesiology.* 2023; 23(1): 47.

19. Patil R, Jaiswal G, Gupta TK. Gunshot wound causing complete spinal cord injury without mechanical violation of spinal axis: Case report with review of literature. *J Craniovertebr Junction Spine.* 2015; 6(4): 149-157.

20. Nakamura IB, Silva MT, Garcia LP, Galvao TF. Prevalence of Physical Violence Against Brazilian Women: Systematic Review and Meta-Analysis. *Trauma, Violence, & Abuse.* 2023; 24(2): 329-339.

21. Wonca International Classification Committee (WICC). *International Classification of Primary Care – 2nd Edition*. 2003. Available from: <https://www.who.int/standards/classifications/other-classifications/international-classification-of-primary-care>

22. World Health Organization. *International Statistical Classification of Diseases and Related Health Problems*. 1994. Available from: <https://icd.who.int/browse10/2019/en>

23. Sun S, Savitz DA, Wellenius GA. Changes in Adverse Pregnancy Outcomes Associated With the COVID-19 Pandemic in the United States. *JAMA Netw Open.* 2021; 4(10): e2129560.

24. de Fatima Marinho de Souza M, Gawryszewski VP, Orduñez P, Sanhueza A, Espinal MA. Cardiovascular disease mortality in the Americas: current trends and disparities. *Heart.* 2012; 98(16): 1207-1212.

25. Dugan J, Shubrook J. International Classification of Diseases, 10th Revision, Coding for Diabetes. *Clinical Diabetes.* 2017; 35(4): 232-238.

26. Suissa K, Schneeweiss S, Lin KJ, Brill G, Kim SC, Patorno E. Validation of obesity-related diagnosis codes in claims data. *Diabetes, Obesity & Metabolism.* 2021; 23(12): 2623-2631.

27. Ho YA, Rahurkar S, Tao G, Patel CG, Arno JN, Wang J, et al. Validation of International Classification of Diseases, Tenth Revision, Clinical Modification Codes for Identifying Cases of Chlamydia and Gonorrhea. *Sexually Transmitted Diseases.* 2021; 48(5): 335-340.

28. Glassgow AE, Wilder J, Caskey R, Munoz G, Van Voorhees B, Kim S. Mental Health Diagnoses among Children and Adolescents with Chronic Medical Conditions in a Large Urban Cohort. *Journal of Behavioral Health Services and Research.* 2020; 9(4): 1-8.
